# Independent origin of genomic imprinting in placental fish

**DOI:** 10.1126/sciadv.aeh5487

**Published:** 2026-11-04

**Authors:** Ying Zhang, Polly Campbell, Alec Kingsley, Yuridia Reynoso, Yuan Kang, Chen-Che Jeff Huang, John H. Werren, David Reznick, Xu Wang

**Affiliations:** ^1^ Department of Pathobiology, College of Veterinary Medicine, Auburn University, Auburn, AL 36849, USA.; ^2^ Department of Evolution, Ecology and Organismal Biology, University of California, Riverside, Riverside, CA 92521, USA.; ^3^ Department of Biology, University of Rochester, Rochester, NY 14627, USA.; ^4^ Department of Anatomy, Physiology & Pharmacology, College of Veterinary Medicine, Auburn University, Auburn, AL 36849, USA.; ^5^ Center for Advanced Science, Innovation and Commerce, Alabama Agricultural Experiment Station, Auburn, AL 36849, USA.; ^6^ HudsonAlpha Institute for Biotechnology, Huntsville, AL 35806, USA.

## Abstract

Genomic imprinting results in biased expression of one parental allele. Such non-Mendelian expression occurs in angiosperms and placental mammals, which share having an active paternal genome during maternal offspring provisioning. It is hypothesized that imprinting modulates conflict between maternal control of resource allocation and paternal control of resource acquisition. If true, then imprinting should evolve in any organism that evolves a placenta. Here, we demonstrate genomic imprinting in a placental poeciliid fish, *Poeciliopsis prolifica*. Using transcriptomes from reciprocal crosses between two inbred strains, we identified 84 protein-coding genes with robust evidence of imprinting in *P. prolifica* placenta. These include the teleost ortholog of mammalian imprinted gene, *grb10*, and a pair of adjacent genes, *dpp4* and *gcga*, with opposing effects on glucose metabolism that are oppositely imprinted in a fashion that potentially resolves parental conflict. Significantly elevated allelic DNA methylation on the promoters of two focal imprinted genes suggests epigenetic mechanisms of imprinting similar to those in mammals. We also discovered the convergent recruitment of a gene network associated with nutrient transport in mammals. To our knowledge, this is the first demonstration of genomic imprinting in a non-mammalian vertebrate and supports the potential role of imprinting in resolving parental conflict.

## INTRODUCTION

The virtues of diploidy include protection against somatic mutations, retention of genetic variation, and enhanced recombination. Virtually all multicellular organisms with diverse, specialized types of cells have life cycles dominated by diploidy, with the haploid phase of the life cycle restricted to gametes. Given this dominance of diploidy, the evolution of genomic imprinting – gene- and tissue-specific epigenetic suppression of either the maternally- or paternally-inherited alleles – requires explanation.

Holman and Kokko (*1*) cite thirteen evolutionary hypotheses for the origin of imprinting. The best supported is the kinship theory of genomic conflict (*2*–*4*), which adopts Hamilton’s (*5*) concept of inclusive fitness to explain the origins of conflict. Mothers are equally related to all of their offspring, but offspring are more related to themselves than to their siblings, particularly when siblings have different fathers. The benefit an offspring gains when acquiring more resources from its mother is associated with a cost to its siblings that is discounted by the probability that they share a given allele (0.5 for a full sibling or 0.25 for a half sibling). Consequently, it is optimal for mothers to allocate equal amounts of resources to each offspring but for each offspring to acquire somewhat more than is in the best interest of the mother to provide, particularly when there is multiple paternity. This inequality is predicted to lead to a perpetual “tug of war” between the maternally- and paternally-inherited alleles in developing offspring, in which selection favors maternal control over allocation at the same time that it favors paternal control over acquisition (*4*). Under the kinship theory (*3*), genomic imprinting is the outcome of selection on paternally-expressed genes (PEGs) to enhance resource acquisition from the mother, and selection on maternally-expressed genes (MEGs) to maintain control of resource allocation.

Genomic imprinting is well characterized in therian mammals and angiosperms (flowering plants). What these two very different groups of organisms have in common is a structure that mediates resource transfer and is derived from both parental genomes – the placental trophoblast in mammals and the endosperm in flowering plants (*6*, *7*). The convergent evolution of genomic imprinting in mammals and flowering plants and the enrichment of imprinted gene expression in the placenta and endosperm suggest that imprinting plays a role in balancing the genetic conflict of interest between the parents (*4*). Previous efforts to identify imprinted genes outside therian mammals failed to discover canonical genomic imprinting: Orthologs of mammalian imprinted genes exhibit biallelic expression in the egg-laying monotremes (*8*), birds (*9*), and a placental lizard (*Pseudemoia entrecasteauxii*) (*10*), suggesting the absence of therian-like imprinting. Extension of the kinship theory to colony level conflict in eusocial hymenopterans (*11*, *12*) motivated searches for genomic imprinting in bees (*13*, *14*). Recent studies provide robust evidence for parent-biased allelic expression in bumblebees (*15*) and European honeybees (*16*). However, whether these expression patterns are mechanistically analogous to genomic imprinting in angiosperms and mammals is a topic of ongoing debate (*17*, *18*).

An independent origin of a placenta would be ideal for investigating whether the evolution of imprinting is indeed driven by the evolution of maternal provisioning via a placenta. Fortunately, nature has done this experiment. Placentation has independently evolved at least nine times in the fish family Poeciliidae (*19*, *20*). The maternal follicle is the maternal side of the placenta in all species examined to date (*21*). The externalized pericardial membrane and yolk sac are the embryonic contributions to the placenta (*21*, *22*).

Here, we test for parent-of-origin gene expression in the black-stripe livebearer, *Poeciliopsis prolifica,* which represents one of the nine origins of placentation in the Poeciliidae. *P. prolifica* offspring weight at birth is seven times the dry mass of the egg at fertilization as a consequence of maternal provisioning during development (*23*, *24*) (fig. S1). We generated two inbred lines (A and B) through multi-generation sib-sib mating (Fig. 1A; Materials and Methods), performed reciprocal (A × B, B × A) and control (A × A, B × B) crosses, and used between-line single nucleotide polymorphisms (SNPs) to measure allelic expression in embryonic and placental tissues from their hybrids. Support for imprinted gene expression requires that allelic expression be highly biased towards the same parent in both directions of the cross. The reciprocal cross design eliminates other possible causes of biased allele representation, such as *cis*-regulatory variation between the two lines or the random silencing of one allele.

**Fig. 1.**
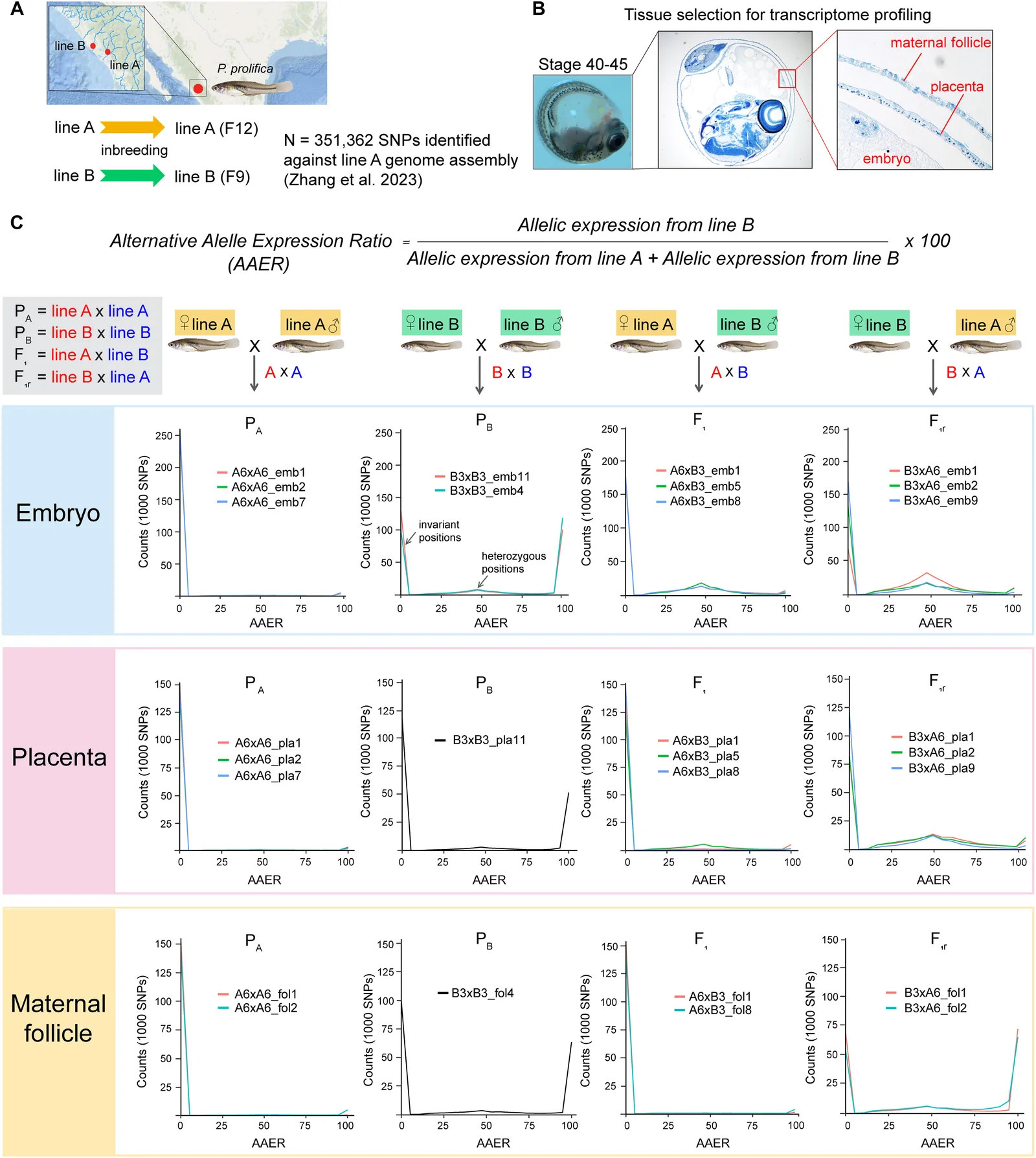
Experimental framework and transcriptome-wide allele specific gene expression profiling in *Poeciliopsis prolifica*. (**A**) Development of semi-inbred lines and exonic SNP discovery. Collection locations of line A and line B within the native range of *P. prolifica* are indicated on the map. Each line was inbred for at least nine generations (line A, F12; line B, F9). RNA-seq reads were mapped to the line A reference genome (*73*) to identify 351,362 high quality informative SNPs used for allele-specific analyses. This simplified geographic map is reproduced from a more comprehensive overview presented in fig. S1A, which provides the entire geographic context. (**B**) Tissue sampling strategy. Late-eyed embryos (developmental stage 40–45) were dissected to isolate three compartments for transcriptome profiling: embryo proper (embryo origin), pericardial membrane (placental tissue, origin unclear), and surrounding maternal follicle (maternal origin). The length of the scale bar is 200 μm. The embryo photograph and histological images are reproduced from fig. S1, which also includes a comparison with the closely related nonplacental species *Poeciliopsis infans*. (**C**) Cross design, definition of the Alternative Allele Expression Ratio (AAER), and genome-wide allelic expression profiles. AAER is defined as the transcriptome read counts from line B, divided by the total read counts from line A plus line B. Four breeding schemes were analyzed in this study: P_A_ (♀ line A × ♂ line A, line A parental cross), P_B_ (♀ line B × ♂ line B, line B parental cross), F_1_ (♀ line A × ♂ line B, F_1_ cross), and F_1r_ (♀ line B × ♂ line A, F_1_ reciprocal cross). The 12 panels of line plots show the distribution of AAER values for informative SNPs in the four crosses in embryo, placenta, and maternal follicle RNA-seq data. In all tissues, parental crosses are dominated by homozygous loci (peaks at 0 or 1). In F_1_ embryo and placenta, AAER values at heterozygous loci center at 0.5, demonstrating primarily biallelic expression, whereas the maternal follicle retains monoallelic (maternal) expression, resembling the parental crosses. Biological replicates per tissue are plotted in color-coded lines.

## RESULTS

### Allele specific expression in the placenta

We used transcriptomes derived from the embryonic yolk-pericardial sac (hereafter, placenta), the whole body of advanced embryos, juvenile fish, and the maternal follicle (table S1), to identify informative SNPs for measurement of allele specific expression (ASE) in reciprocal crosses between *P. prolifica* lines A and B (Fig. 1, B and C). Across all tissues, we identified a total of 351,362 SNPs covering 24,225 genes with an average of 13 SNPs/gene. Thus, our SNP-based coverage was truly transcriptome-wide. ASE-informative SNPs are those that correspond to fixed differences between strains A and B. To identify ASE-informative SNPs and confirm the accuracy of our dissections, we classified high coverage (see Methods) SNPs according to tissue. Of 272,496 and 193,524 SNPs in between-line (F_1_ and F_1r_) embryo body and placenta transcriptomes, respectively, 104,232 and 55,224 were homozygous for alternative alleles in all three individuals from each within-line cross (P_A_ and P_B_) (Fig. 1C). Most of these had an alternative allele expression ratio (AAER) of 0.5, consistent with equal expression from both parental alleles as expected for most genes (Fig. 1C). The remaining SNPs represented residual heterozygous sites in line B and were excluded from further analyses. Importantly, the distribution of AAER in placenta is similar to that in embryo whole body, indicating that embryo and placenta share the same genotype (Fig. 1C and data S1 and S2). In maternal follicle transcriptomes, the distribution of AAER in line A females crossed to line B males is similar to that in females used in A × A crosses and the same is true for line B females (Fig. 1C and data S3). These results demonstrate that SNP differences between lines A and B are sufficient to measure ASE for a large number of genes and confirm that the placenta is embryo-derived and is distinct from the maternally-derived follicle.

### Widespread parental bias in the placenta

Imprinted gene expression is highly enriched in the mammalian trophoblast, the embryo-derived part of the placenta (*25*, *26*). If the evolution of genomic imprinting is driven by parental conflict over maternal prenatal provisioning, then we expect similar enrichment in the *P. prolifica* placenta. To test this prediction, we initially filtered for transcripts with at least two line-specific SNPs and AAER >0.65 in one direction of the inter-line cross and < 0.35 in the other (see Methods). Allele expression ratio cut-offs were chosen based on our prior work on genomic imprinting in mammals (*27*, *28*) and recognition that the minority of mammalian imprinted genes have truly monoallelic expression with the majority characterized by strong parent-of-origin allelic bias (*29*). This approach identified 650 maternally biased transcripts and 73 paternally biased transcripts (Fig. 2A) with an average of 2.70 (±SD 1.47) SNPs per transcript (data S4). The excess of maternally biased genes cannot be explained by maternal tissue contamination. Unlike the maternal decidua and embryo-derived trophoblast in placental mammals, the follicle and yolk-pericardial sac in placental poeciliids are not physically integrated (Fig. 1B). We cannot rule out the possibility that a small fraction of maternally biased genes are the undegraded transcripts of maternal-effect genes deposited in the egg prior to fertilization. However, given that known maternal effect genes in zebrafish do not act beyond specification of the embryonic body plan [e.g., (*30*, *31*)], we would not expect maternal transcripts to persist at the late developmental stage studied here. Maternally biased genes were enriched for functions in ion transport, signaling, development, and morphogenesis (Fig. 2B); paternally biased genes were highly enriched for terms associated with muscle development and differentiation (Fig. 2C). The contrasting enrichment profiles of maternally- and paternally-biased genes highlight parental effects on distinct developmental processes but collectively overlap with enriched functions for imprinted and co-expressed biallelic genes in mouse (*32*).

**Fig. 2.**
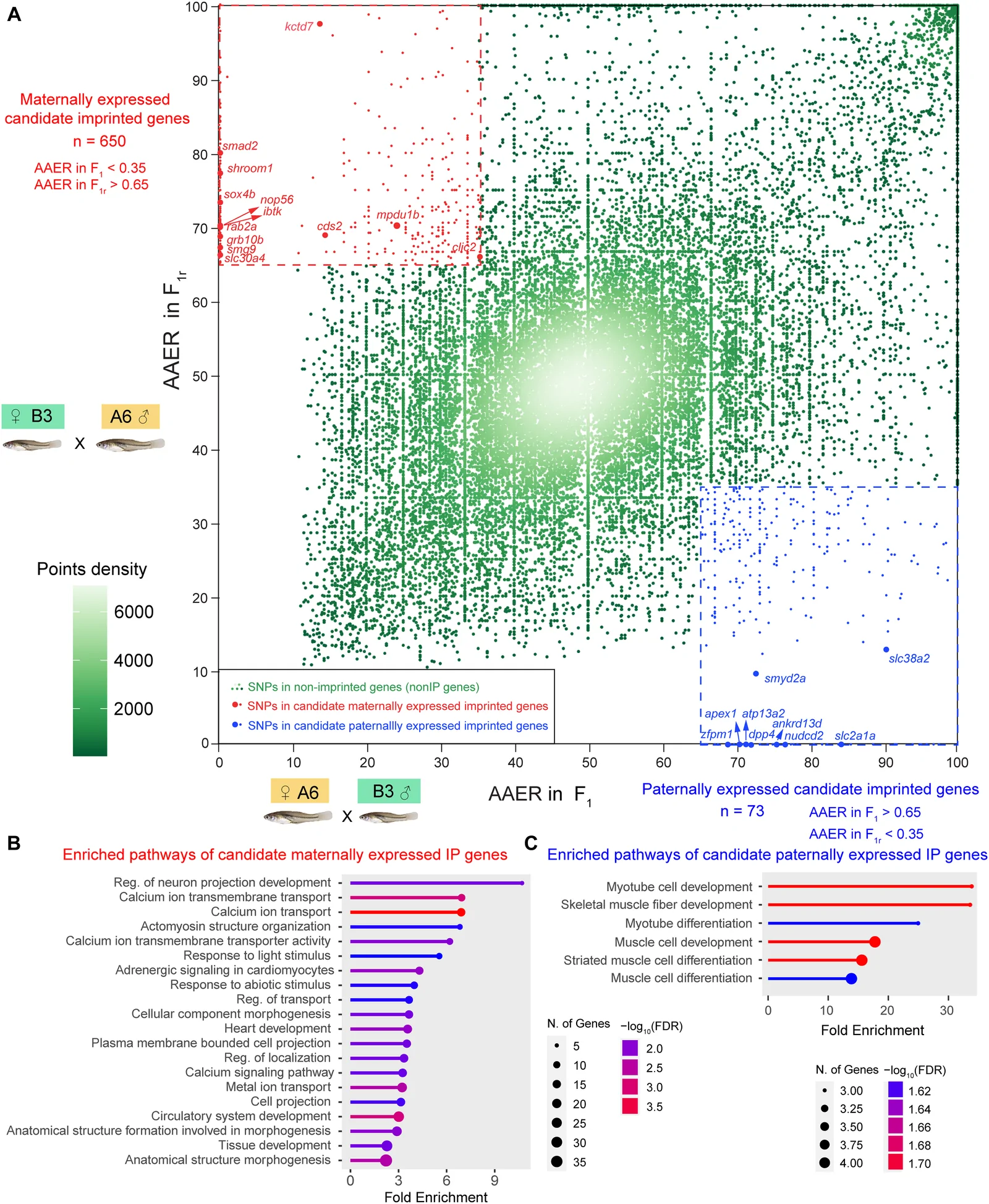
Reciprocal cross analysis identifies hundreds of genes with parent of origin allelic bias in *Poeciliopsis prolifica* placenta. (**A**) Each point in the scatterplot represents a single heterozygous SNP assayed in placenta (pericardial membrane) with RNA-seq from the two reciprocal crosses: ♀ A6 × ♂ B3 (F_1_; horizontal axis) and ♀ B3 × ♂ A6 (F_1r_; vertical axis). The Alternative Allele Expression Ratio (AAER) is defined as in Fig. 1. Density-shaded green dots represent SNPs in genes showing biallelic expression, classified as non-imprinted. SNPs in maternally biased candidate imprinted genes are represented using red dots (upper-left dashed box), with selection criteria of AAER <0.35 in F_1_ and AAER >0.65 in F_1r_. SNPs in paternally biased candidate imprinted genes are represented using blue dots (bottom-right dashed box), with selection criteria of AAER >0.65 in F_1_ and AAER <0.35 in F_1r_. The names of candidate imprinted genes involved in ion transport and tissue development are indicated. (**B**) Over-represented pathways among maternally biased candidate imprinted (IP) genes. Gene Ontology (GO) analysis of the 650 maternally biased loci reveals significant enrichment (false-discovery rate, FDR < 0.05) for pathways linked to ion transport and embryonic tissue development. (**C**) Over-represented pathways among paternally biased candidate IP genes. GO analysis of the 73 paternally biased loci reveals significant enrichment (FDR < 0.05) for pathways linked to development and differentiation of muscle. For both panel B and C, bar length represents fold enrichment, dot size scales with the number of genes in each pathway, and color intensity denotes enrichment significance quantified by -log_10_ FDR.

### Convergent genomic imprinting with humans

To increase stringency and reduce false positives, we extracted the maternally- and paternally-biased genes for which there were significant differences in allelic expression in both cross directions (*28*) (see Materials and Methods). Seventy-one maternally-biased genes (55 protein-coding) and 35 paternally-biased genes (29 protein-coding) met this criterion (Fig. 3A, fig. S2, and tables S2 and S3). These genes are strong candidates for genomic imprinting in *P. prolifica* placenta. Among protein-coding genes, only 18% of placental MEGs (10/55) and 14% of placental PEGs (4/29) met our criteria for imprinted expression in embryonic tissue (Fig. 3A). The AAERs of the remaining genes in either reciprocal line ranged from 0.35 to 0.65, exceeding our imprinting thresholds (Fig. 3A), and including eight placental imprinted genes that exhibited reversed allelic expression patterns in whole embryos relative to placental ratios (data S4).

**Fig. 3.**
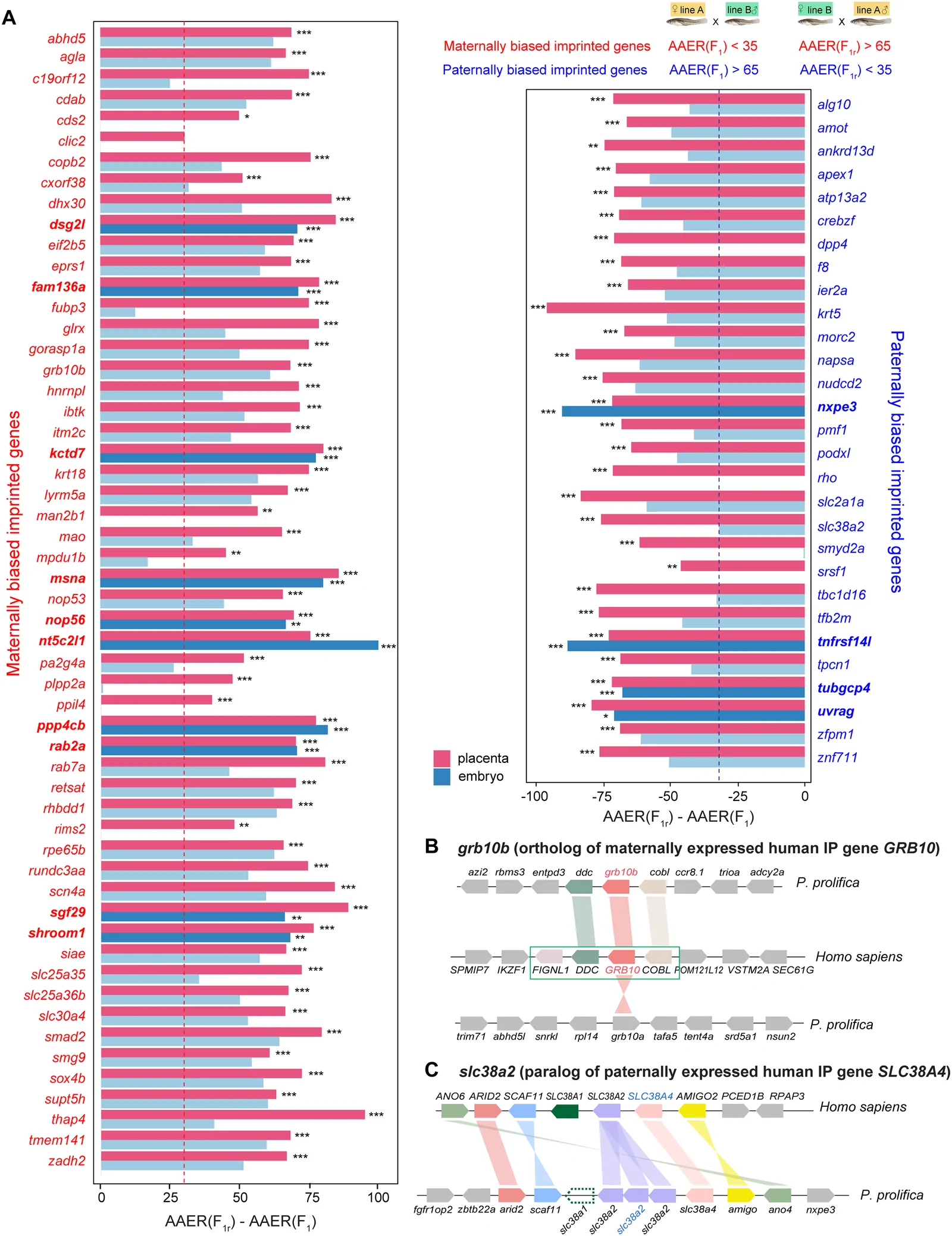
High confidence candidate imprinted genes in the *Poeciliopsis prolifica* placenta and synteny analyses of candidates that are also imprinted in human placenta. (**A**) Barplot of degree of bias (AAER in F_1r_ - AAER in F_1_) for statistically supported protein-coding imprinted genes in placenta (*P* < 0.05, Fisher’s exact test): 55 maternally expressed IP genes (red font, left panels) and 29 paternally expressed IP genes (blue font, right panels). The degree of bias of each gene is presented in both placenta (red) and embryo (blue). Genes without allelic bias in the embryo are shown in light blue and those with allelic bias are shown in dark blue; genes not expressed in the embryo are blank. The genes imprinted in both tissues are highlighted in bold. Statistical significance for each gene is indicated beside the bars (*P < 0.05, **P < 0.01, ***P < 0.001). The grey dashed line marks 30% allelic bias. (**B**) Conserved microsynteny of human-fish *GRB10*-*grb10b* imprinting domain. Comparative gene neighborhood analysis confirmed that *grb10b*, not *grb10a*, is syntenic to mammalian *GRB10*. The boxed *DDC*-*GRB10*/*grb10b*-*COBL* segment delineates the conserved core shared by teleosts and mammals. (**C**) The human duplicated gene cluster *SLC38A1-A2-A4* preserves gene neighborhood collinearity with its *P. prolifica* counterpart *slc38a2-a2-a4*.

Notably, two of our candidate imprinted genes are either orthologs or paralogs of imprinted genes with the same parental allelic bias in placental mammals. *GRB10* (growth factor receptor bound protein 10) is a well-characterized imprinted gene that is maternally expressed in both human and mouse placenta, where it mediates glucose metabolism and acts as a brake on placental and fetal growth (*33*, *34*). There are two copies of this gene in *P. prolifica* (*grb10a* and *grb10b*), presumably due to ancient whole-genome duplication events in teleost ancestors. Only *grb10b* is imprinted in *P. prolifica* (Fig. 3A). Using gene neighborhood analysis (Methods), we confirmed that *grb10b*, not *grb10a*, is syntenic with *GRB10* in the human genome (Fig. 3B). Similarly, *SLC38A4* (solute carrier family 38, member 4) is a paternally expressed growth-promoting amino acid transporter in human and mouse placenta (*35*). Microsynteny analysis at the *SLC38A4* locus between human and *P. prolifica* revealed an ancient tandem amplification of *SLC38A1-A2-A4* with conserved collinearity. Following the teleost-specific third round of whole-genome duplication, *SLC38A1* was lost in most teleosts (fig. S3), whereas *P. prolifica* experienced a tandem duplication of *slc38a2* (Fig. 3C and fig. S3). Of the three adjacent *slc38a2* copies, only the middle copy exhibited paternal allele-biased expression and was identified as an imprinted gene (Fig. 3A), suggesting that imprinting evolved after the tandem duplication and became restricted to a single paralog in the placenta. Placenta-specific knockdown of *Slc38a2* in mouse reduces placental and fetal weight (*35*, *36*), suggesting that *Slc38a4* and *Slc38a2* retain similar functions. Taken together, these results are consistent with convergent evolution of imprinted expression at the *Slc38a* locus in placental mammals and fish. Three other imprinting candidates in *P. prolifica* belong to gene families that include mammalian imprinted genes: a tumor necrosis factor receptor (*tnfrsf14l*) and two zinc finger proteins (*zfpm1, znf711*) (*37*). Collectively, these results indicate that the evolution of imprinting in *P. prolifica* included independent recruitment of genes or gene family members that are also imprinted in mammals.

### Convergent recruitment of *dpp4* network

Among high confidence imprinted genes, *dpp4* (dipeptidylpeptidase 4) stood out as the most significant of >2,000 upregulated genes in *P. prolifica* placenta relative to embryo body (Fig. 4, table S4, and data S5). Consistent with the strong paternal bias for *dpp4* in the RNAseq data (F_1_ = 100%, F_1r_ = 71%), pyrosequencing validation indicated 91% and 81% paternal allelic expression in the F_1_ and F_1r_ crosses, respectively (Fig. 4E and table S5; see fig. S4 for additional validations). Whereas *dpp4* is not imprinted in mammals, it is expressed in the placentas of a range of therian species [e.g., opossum, armadillo, dog, human (*38*)], suggesting recruitment to placental function early in the evolution of the mammalian placenta. We therefore used evolutionary rate correlation analysis (*39*, *40*) (see Materials and Methods) in mammals to identify the set of genes that coevolve with *dpp4* and check for overlap with genes of interest in *P. prolifica* placenta. For the analysis, we generated a reference list of orthologs shared between the ERC mammalian dataset and *P. prolifica* (data S6). The *DPP4* reciprocal rank network is shown in Fig. 5A. Strikingly, among the top 15 *DPP4* reciprocal ERC partners in mammals, 9 are upregulated in *P. prolifica* placenta relative to embryo (>2-fold increase, adjusted *P* < 0.05), and 3 are candidate MEGs (Fig. 5A). Furthermore, 4 of these placenta-upregulated genes, including one candidate MEG, are in *DPP4*’s top 5 ERC network (>4-fold increase in placenta vs. embryo, *P* < 0.001; Fig. 5B). The robust expression (TPM > 5) of 10 of the top 15 *DPP4* reciprocal ERC partners in human placenta (fig. S5) confirms a placental function for many of these genes in mammals. These results suggest that *dpp4* and its conserved core set of interacting genes were independently recruited in mammals and the Poeciliidae during the evolution of the placenta.

**Fig. 4.**
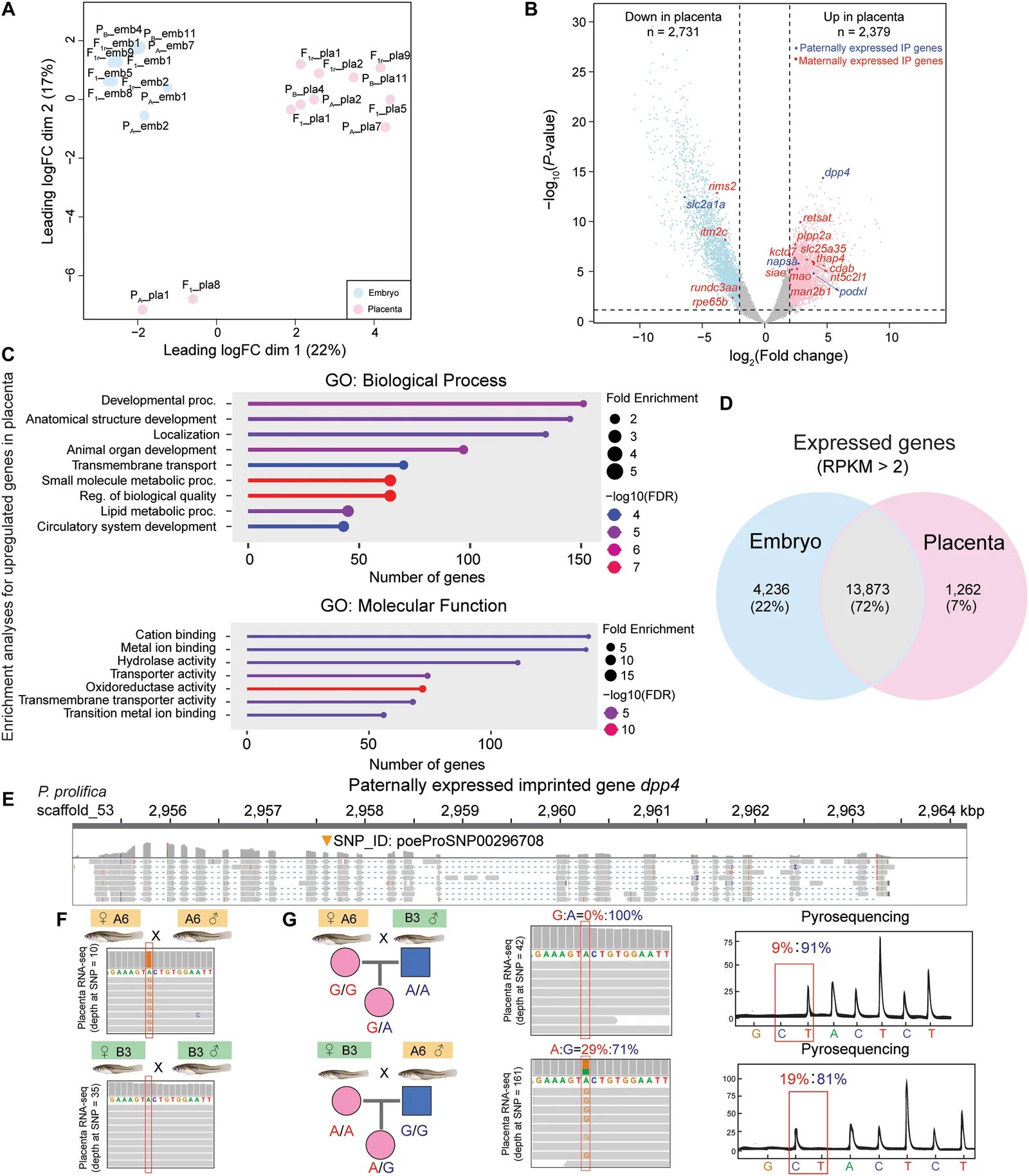
Transcriptome divergence between embryo and placental tissues, placenta-upregulated gene enrichment in *Poeciliopsis prolifica*, and pyrosequencing validation of paternally biased imprinted gene *dpp4*. (**A**) Multidimensional scaling (MDS) plot of the RNA-seq expression data in embryos (blue) and placental samples (pink). (**B**) Volcano plot comparing the embryo transcriptome to the placental. Differentially expressed genes (DEGs) that are upregulated (*right*, n = 2379) and downregulated (*left*, n = 2731) in the placenta are determined based on a cutoff of FDR < 0.05 and log_2_(fold-change) > 2. Statistically significant imprinted genes in the placenta are highlighted in red for maternally biased loci and blue for paternally biased loci. *Dpp4*, a paternally expressed imprinted gene identified in this study, represents the most significantly upregulated DEG in the placenta. (**C**) Gene ontology (GO) enrichment analyses of significantly upregulated genes in the placenta compared to the embryo (log_2_(fold-change) > 2 and FDR < 0.001). Bar length represents the number of genes, dot size scales with fold enrichment, and color intensity reflects enrichment significance quantified by -log_10_(FDR). (**D**) Venn diagram shows the number of expressed genes (RPKM >2) in both the embryo and placenta. (**E** to **G**) Validation of imprinting at the *dpp4* locus. (E) Integrative Genomics Viewer (IGV) pile-up of placental RNA-seq reads aligned to *dpp4* gene region. Genome location of the informative exonic SNP is indicated by the orange arrow. (F) RNA-seq alignments in the semi-inbred parental crosses. (G) Pyrosequencing validation in reciprocal F1 crosses. **Left**: pedigrees illustrating the cross design (paternal allele is labeled in blue and maternal is labeled in pink). **Middle**: differential allelic expression inferred from RNA-seq data. **Right**: pyrosequencing validation of differential allelic expression showing the pyrograms.

**Fig. 5.**
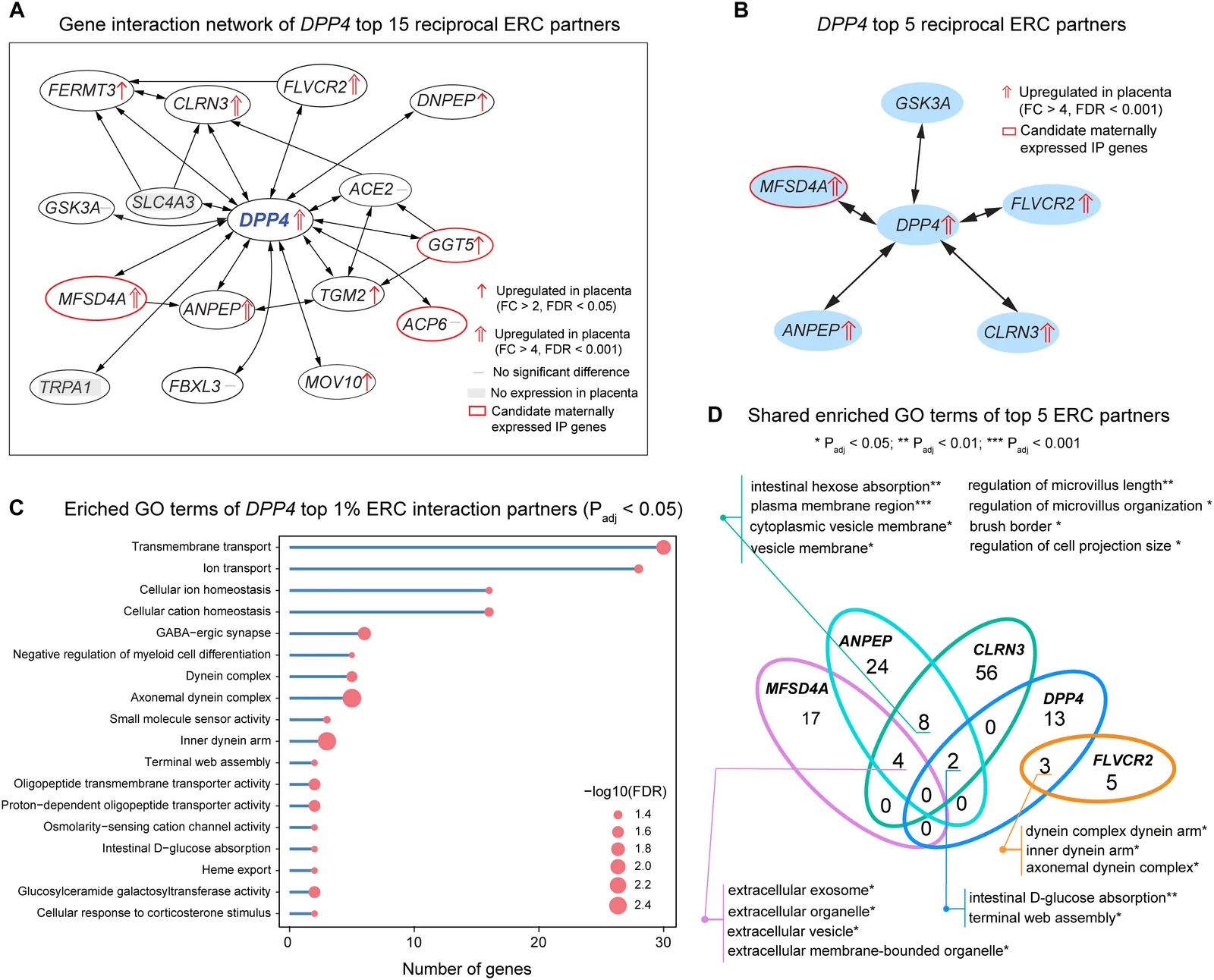
Evolutionary rate correlation analysis identifies *DPP4* as an evolutionary hub gene in mammals, with ERC interaction partners enriched for functions in nutrient absorption in intestinal epithelial cells. (**A**) Mammalian *DPP4* ERC reciprocal interaction network for top 15 partners among overlapping mammal-*Poeciliopsis* orthologs (*40*). Nodes represent genes, with *DPP4* shown at the center. Red arrows denote upregulation in *P. prolifica* placenta relative to embryo at two fold-change thresholds (FDR < 0.05). Red-outlined genes are candidate maternally expressed imprinted (IP) genes in the *P. prolifica* placenta. (**B**) *DPP4* top five reciprocal ERC partners. Expression status in *P. prolifica* placenta and candidate maternal IP genes are highlighted as in panel A. (**C**) Barplots showing enriched Gene Ontology (GO) terms among the top 1% *DPP4* mammalian ERC partners (*n* = 100 genes, FDR < 0.05). (**D**) Shared enriched GO terms among *DPP4* and reciprocal ERC partners identified in panel B that are also upregulated in *P. prolifica* placenta. Overlapping regions indicate GO terms jointly enriched across genes (*P*_*adj*_ < 0.05).

### Placental co-option of gut genes

Novel organs evolve via co-option of genes already expressed in the tissue, recruitment of genes expressed in other tissues, and de novo origin of new genes (*41*, *42*). Several lines of evidence suggest that *dpp4* and its coevolving interaction partners were recruited from the gut interaction networks to the placenta in poeciliid fish and mammals. In humans, *DPP4* and 9 of its top 15 ERC partners are expressed in both intestine and placenta (Human Protein Atlas (*43*), Fig. 6 and fig. S5), two tissues with specialized functions in absorption and nutrient transport. Using zebrafish as a representative non-placental teleost, we found that *dpp4* is highly expressed in intestine and digestive tract relative to all other tissues (*44*, *45*) (Fig. 6C), suggesting conservation of function in the gut. Furthermore, across mammals, the top 1% of *dpp4* coevolving interaction partners is enriched for transmembrane transport (adjusted *P* = 0.04, Fig. 5C) and 2 of the 5 genes (*anpep* and *clrn3*) with the strongest signal of coevolution with *dpp4* share multiple enrichment terms associated with intestinal structure and function (Fig. 5D and data S7). Robust upregulation of *anpep* and *clrn3* in *P. prolifica* placenta relative to embryo body is consistent with collective recruitment of *dpp4* and interacting genes to the placenta. Taken together, these results suggest that the evolution of a placenta from non-homologous tissues in poeciliids and mammals involved convergent recruitment of a conserved *dpp4* gene network associated with nutrient transport in the gut, a tissue inherited from a common eumetazoan ancestor (*41*, *46*), to fulfill a similar function in the placenta.

**Fig. 6.**
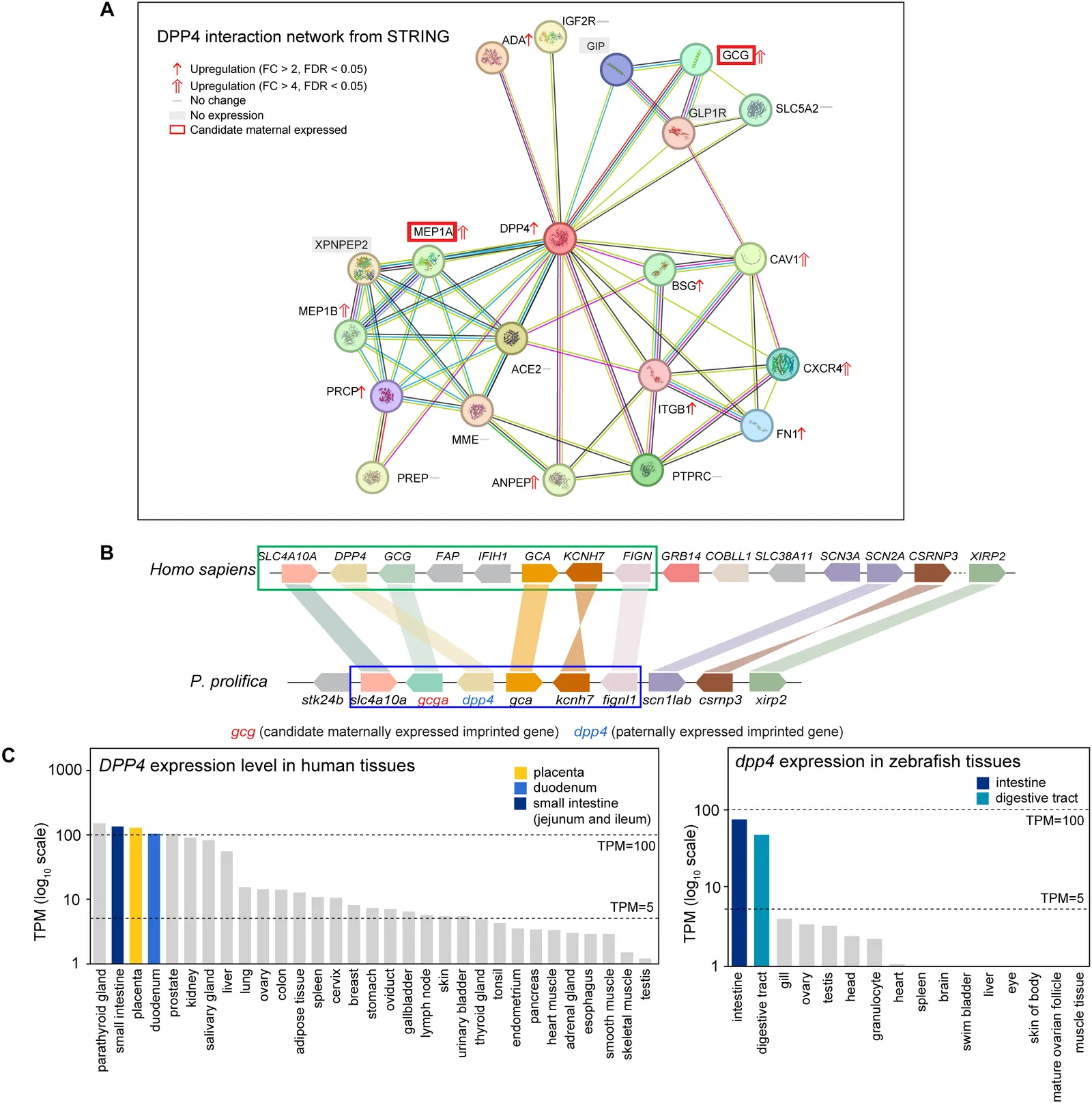
*DPP4* protein interaction partners, gene expression landscape, and genomic synteny in human and *Poeciliopsis prolifica*. (**A**) The DPP4 protein interaction network in humans, derived from STRING database (version 12.0), identifies *DPP4* as a conserved and highly connected molecular hub. Upregulation in the *Poeciliopsis prolifica* placenta relative to embryo, together with imprinting status, are indicated for orthologous genes in this plot. (**B**) Conserved microsynteny between human and *P. prolifica* in the gene neighborhood of paternally expressed imprinted gene, *dpp4*. The immediately adjacent gene, *gcg-a*, is a candidate maternally expressed imprinted gene in *P. prolifica*, mirroring mammalian imprinting clusters characterized by opposing imprinting directions. (**C**) *DPP4* expression level across human tissues (placenta, yellow, gut tissues, blue; data from Human Protein Atlas, left), and tissue specific expression profile of *dpp4* in zebrafish (gut tissues, blue; data from https://www.bgee.org/, right).

In human placenta, *DPP4* expression is enriched in trophoblast cell types critical to uterine invasion in the first trimester (*47*–*49*) and to maternal-fetal exchange and signaling in later gestation (*38*, *50*). Merged analysis of single-cell RNA sequencing data from two normal first-trimester placentas (*49*) and eight normal term placentas (*49*, *50*) confirmed these patterns (fig. S6). Notably, 11 of the top 20 DPP4-interacting proteins in humans are upregulated in *P. prolifica* placenta relative to embryo and two (*GCG*, glucagon, and *MEP1A*, meprin 1 alpha) are candidate MEGs (Fig. 6A and fig. S7). Moreover, 10 of the 11 genes that are upregulated in *P. prolifica* placenta are also expressed in human and/or mouse placenta (*51*, *52*), including 4 genes for which placenta is the tissue with the first or second highest expression as compared to 27 other human tissues (*PRCP*, *FN1*, *CAV1*, *ITGB1*; fig. S7). These results echo the overlap between mammalian *Dpp4* ERC partner genes and candidate imprinted and/or upregulated genes in *P. prolifica* placenta, enforcing the notion of convergent evolution in placental fish and mammals.

### *dpp4*-*gcg*: candidate conflict mediators

*DPP4* and *GCG* are neighboring genes on human chromosome 2. This relationship is conserved in the *P. prolifica* genome (Fig. 6B). Two orthologs of *gcg* (*53*) were found in the *P. prolifica* genome, *gcga* and *gcgb* (fig. S8). *gcga* is located immediately adjacent to *dpp4* and encodes a protein of 178 amino acids (aa), which is comparable in length to the human *GCG* (180aa), whereas *gcgb* encodes a much shorter protein (123 aa) lacking a C terminus domain (fig. S8). The top three most highly expressed tissues for human *GCG* are pancreas, small intestine, and colon (fig. S8). In zebrafish, *gcga* is most highly expressed in the spleen, intestine, and digestive tract (fig. S8), while *gcgb* is predominantly expressed in larvae, the digestive tract, and intestine (fig. S8). Notably, *gcga* expression in the intestine and digestive tract is approximately 2-fold higher than that of *gcgb* (fig. S8). Together with its conserved synteny with *dpp4*, greater protein similarity to human *GCG*, and significantly higher expression in the intestine and digestive tract, our findings suggest that *gcga* more closely resembles human *GCG* in its biological function than does *gcgb*.

The newly discovered *dpp4*-*gcga* imprinting cluster is intriguing for two reasons. First, clustering in the genome is a hallmark feature of canonical mammalian imprinted genes that facilitates shared regulatory control of allelic expression within the cluster (*54*). The proximity of a PEG and candidate MEG in the *P. prolifica* genome raises the possibility that similar regulatory architecture exists in placental fish. Second, in mammals, placental *Dpp4* and the bioactive product of *Gcg*, glucagon-like peptide 1 (Glp-1), are opposing regulators of glucose metabolism [reviewed in (*55*)]. In the placenta, Glp-1 is a negative regulator of nutrient transport and thus fetal growth (*56*) whereas *Dpp4* keeps Glp-1 activity in check by inactivating the protein (*57*). In humans, enzymatically competent DPP4 is released into maternal blood stream in extracellular vesicles derived from the placenta and is thought to regulate maternal metabolism (*58*). Opposing effects of placental gene products and placental modulation of maternal physiology are proposed signatures of parental conflict over maternal resource allocation that favor the evolution of genomic imprinting (*2*, *4*, *59*, *60*). These features, together with the direction of parental allelic bias of *dpp4* and *gcg*, identify these genes as candidate mediators of conflict between the maternally and paternally inherited genome over maternal resource allocation in the *P. prolifica* placenta.

### Allele-specific methylation is consistent with epigenetic regulation of imprinting

We performed PacBio HiFi sequencing to investigate allele-specific DNA methylation patterns. A total of 12 Gb of HiFi sequence data were generated, corresponding to approximately 18× genome coverage. We inferred CpG methylation directly from the HiFi reads using the DNA polymerase kinetics recorded during sequencing. Long-read sequencing further enabled haplotype-resolved allele-specific methylation analysis by assigning methylation calls to individual alleles using informative SNPs. In paternally expressed *dpp4*, we used a T/C SNP located adjacent to the promoter CpG island to partition the PacBio reads into two haplotypes (fig. S9). Four CpG sites within the CpG island were covered by phased reads and were used for allele-specific methylation analysis. Across the four sites, methylation on the T allele (81.3%, 14/19 methylated CpGs) was significantly higher than that on the C allele (42.5%, 17/40 methylated CpGs; *P* = 0.016, Fisher’s exact test; fig. S9). We detected similar, significant differential allelic methylation at the *slc38a2* promoter CpG island (*P* = 0.0002; fig. S10). Although the parental origin of the two haplotypes could not be directly assigned, the observed allele-specific methylation is consistent with epigenetic regulation of paternally-biased expression at *dpp4* and *slc38a2*. These findings provide evidence that allele-specific DNA methylation may contribute to the regulation of genomic imprinting in *P. prolifica*.

## DISCUSSION

To our knowledge, our results represent the first unqualified demonstration of genomic imprinting outside of mammals and angiosperms. We favor the proposal that imprinting evolves in response to parental conflict over maternal resource allocation and acquisition because the majority of these genes are uniquely imprinted in the placenta. Furthermore, the pairing of PEG *dpp4* with MEG *gcg* fulfills the conditions of the kinship theory of genomic conflict (*2*) because of their antagonistic interactions in mammals. The protein product of *Gcg* limits placental nutrient transport, and hence potentially limits allocation to the embryo. The product of *Dpp4* negatively regulates the action of *Gcg*, which potentially enhances acquisition by the embryo. This pattern of imprinting matches the opposing functions and direction of parental bias for PEG *Igf2* and MEG *Igf2r* in mammals (*2*).

Multiple lines of evidence suggest some convergence in the evolution of genomic imprinting in the poeciliid and mammalian placenta. Two of our candidate imprinted genes (*grb10* and *slc38a4*) are imprinted with the same parental bias found in humans and mice, and others are members of gene families that include imprinted genes in mammals. Our strongest result pertains to the PEG *dpp4*. There is high overlap between the coevolving partner genes of *Dpp4* in mammals and genes that are imprinted and/or upregulated in *P. prolifica* placenta. This suggests that a core set of interacting genes was independently recruited to placental functions in *P. prolifica* and mammals. Moreover, the strong allelic bias in DNA methylation on *dpp4* and *slc38a2* promoters suggests that at least one epigenetic mechanism of imprinted gene regulation is shared between *P. prolifica* and mammals.

In our earlier paper (*61*), we considered the evolution of gene expression in the maternal contribution to the poeciliid placenta – the follicle. The evolution of a placenta involves transforming the follicle from fully provisioning an egg before fertilization to primarily provisioning developing embryos. Our comparative transcriptomic analysis of two independent origins of placentation in Poeciliidae revealed 86 and 133 upregulated genes in each placental species relative to its non-placental sister species, but only four genes were common to both. In spite of there being little in common in terms of gene identity, there was much in common in the functional pathways represented in the two origins of placental provisioning.

The combined results of the current and past paper reveal different facets of the evolution of complex organs. The association between *dpp4* and *gcg* illustrates one mechanism - the recruitment of pre-existing genes and gene networks for new functions (*41*). *Dpp4* is also highly expressed in the digestive tracts of both humans and zebrafish. This association suggests that its contribution to the transport of nutrients in the intestine was recruited for the provisioning of developing embryos. The evolution of the poeciliid placenta mirrors this pattern of recycling existing networks for new functions at the tissue level. The maternal contribution is a modification of the follicle (*21*) while the embryonic contribution is an externalization of the pericardial membrane and the yolk sac (*22*). Our comparative transcriptomic analysis of the follicle revealed a second mechanism – convergence via the recruitment of different genes with similar collective functions in independent transitions to embryonic provisioning (*61*). This pattern is consistent with the notion that polygenic traits offer multiple pathways to the same endpoint.

This and prior publications on the evolution of the poeciliid placenta [e.g., (*22*, *61*)] collectively illustrate the multifaceted nature of the evolution of novel, complex organs. Pre-existing tissues have been recruited and their functions modified. Pre-existing networks of genes have been recruited and upregulated to fulfill new functions. Imprinting is currently established for just one poeciliid species, but nature has provided us with at least eight more replicates. This replication includes clusters of closely related species with and without placentation. These resources provide an exceptionally promising system for the study of how and why genomic imprinting and placentation evolve.

## MATERIALS AND METHODS

### Description of developing embryos and the placenta

Development is intraovarian with each embryo contained within its own maternal follicle throughout development. The embryo is contained within two concentric layers of tissue, the outer being the follicle and the inner the yolk-pericardial sac (the latter referred to as the “placenta” in our paper). If we adopt Mossman’s definition of the placenta as an apposition of maternal and embryonic tissue devoted to sustaining the developing embryo, then the follicle is the maternal contribution and the yolk-pericardial sac is the embryonic contribution to the placenta (*62*). *P. prolifica* has a smooth follicle and a yolk-pericardial sac of thickness equal to the follicle (*22*). Both layers are well vascularized and are not in direct physical contact with each other (see Fig. 1B and fig. S1). A consequence is that it is easy to separate the follicle, yolk-pericardial sac and embryo without risk of contaminating one tissue type with the others. All of our research dealt with “late eyed embryos” [stages 20 and above; see Tavolga and Rugh (*63*)], which means that the integument is fully developed and fin rudiments or fins with rays are present. Embryos are rapidly increasing in dry mass throughout these stages of development (*64*).

### Fish and dissections

*Poeciliopsis prolifica* (NCBI Taxonomy ID 188132) and *Poeciliopsis infans* (NCBI Taxonomy ID 188128) were collected by D. Reznick. *P. prolifica* line A and line B were derived from fish captured from Rio El Palillo (Sinaloa State, Mexico; see fig. S1A). Lines A and B were each initiated with two pairs of fish from the stock culture and then kept as separate stock cultures for three years. Multiple semi-inbred lines were developed from each stock culture by mating siblings for up to 12 generations (for line A) or 9 generations (for line B) of sib-sib matings. Three collections of *P. infans* were derived from fish collected from Rio Ameca, Rio Santiago, and Rio de la Pasion (Jalisco State, Mexico; fig. S1A). Fish were euthanized using 100 mg/L buffered MS-222 (tricaine methanesulfonate, Syndel Inc., Ferndale, WA, USA). Advanced embryo, maternal follicle and placenta were obtained from *P. prolifica* parental [P_A_ (lineA × lineA), P_B_ (lineB × lineB)] and reciprocal lines [F_1_ (lineA × lineB), F_1r_ (lineB × lineA)]. All samples used are listed in table S1. All procedures were approved by the University of California, Riverside Institutional Animal Care and Use Committee (IACUC) with protocol number 20170006.

### Histological analysis of the matrotrophic species, *Poeciliopsis prolifica*, and the closely related lecithotrophic species, *P. infans*

*P. infans* is the lecithotrophic species most closely related to *P. prolifica* (fig. S1B). Embryos with placental and maternal follicle tissues were fixed in Bouin’s Fixation solution (Ricca Chemical Company, 1120–16) for 2 hours at room temperature with gentle agitation. After fixation, samples were rinsed in 1× PBS 3 times for 10 mins, followed by paraffin embedding and sectioning. Sections (4 μm) were deparaffinized, rehydrated, and then stained with filtered 1% (w/v) toluidine blue solution for 8 min. Excess stain was gently rinsed off with distilled water, followed by counterstaining with 1% (w/v) methylene blue solution for 4 min. Slides were then rinsed, dehydrated, and mounted. Images were obtained using a Nikon E800 microscope (Nikon Inc., Melville, NY, USA). Compared to *P. infans* (Matrotrophy Index, MI = 0.86), *P. prolifica* (MI = 7.0) has a much higher degree of superfetation, in which females typically have three to four developing broods of young, as opposed to one to two broods in *P. infans* (fig. S1, C and D). Matrotrophy and superfetation facilitate more frequent brood production with fewer offspring per brood (*65*). This allows females to distribute their energy relatively evenly to reproduction and to reduce the average energetic demand of locomotion (*66*–*68*). Superfetation is significantly associated with matrotrophy (*19*, *69*), such that the presence of either appears to facilitate the evolution of the other. However, there is no strict link between the two traits. Whereas all species of *Poeciliopsis* inherited superfetation from their common ancestor, matrotrophy evolved independently three times within the genus (*69*). More generally, the distribution of these two traits in the family Poeciliidae reveals that either trait can evolve first (*69*). In our histology analysis, we found that the maternal follicle of *P. prolifica* is thicker than *P. infans* (fig. S1E), which is consistent with the previously reported positive correlation between the degree of matrotrophy and maternal follicle thickness (*70*).

### RNA extraction and sequencing

To investigate patterns of gene expression and identify parentally-biased genes, embryo, placenta, and maternal follicle samples were collected from *P. prolifica* inbred lines A and B, and reciprocal crosses between lines, at advanced developmental stage 40–45 (table S1 and fig. S1E). Total RNA from advanced embryo, maternal follicle, and placenta was extracted using the RNeasy Plus Mini kit (Qiagen, Redwood City, CA, USA), and mRNA-seq libraries were prepared using the TruSeq RNA Library Prep kit v2 (Illumina, San Diego, CA, USA). After checking the size distribution on an Agilent TapeStation 4200 using High-Sensitive D1000 Screen Tape (Agilent Technologies, Santa Clara, CA, USA), the libraries were sequenced using a 2 × 125 bp PE format on an Illumina HiSeq2500 Sequencer at the Genomics Service Laboratory at the HudsonAlpha Institute for Biotechnology (HudsonAlpha Institute for Biotechnology, Huntsville, AL, USA).

### DNA extraction and PacBio HiFi sequencing

High-molecular-weight (HMW) genomic DNA was extracted from *P. prolifica* line A samples using the MagAttract HMW DNA Kit (Qiagen, MD, USA) according to the manufacturer’s instructions. DNA concentration and quality were assessed prior to library preparation, and 6 μg of HMW DNA was used to construct a SMRTbell library. Library preparation and PacBio circular consensus sequencing (CCS) were performed at the HudsonAlpha Genome Sequencing Center (HudsonAlpha Institute for Biotechnology, Huntsville, AL, USA) using the PacBio Revio system with SPRQ chemistry. In addition to generating HiFi reads, DNA methylation information was obtained from the raw sequencing data based on changes in DNA polymerase kinetics during SMRT sequencing, including interpulse duration (IPD) and pulse width (PW) signals.

### Identification and analyses of exonic SNPs between *P. prolifica* lines A and B

Raw sequence data quality was checked with fastQC v0.11.9 (*71*). Trimmomatic v0.39 (*72*) was used to discard low-quality reads, trim adaptor sequences, and eliminate poor-quality bases. Trimmed RNA-seq reads were mapped to the *P. prolifica* reference genome assembly (GCF_027474105.1) (*73*), using both TopHat v2.1.1 (*74*) and STAR version 2.7.5 (*75*) (table S1). Alignment BAM files were merged into a combined BAM using SAMtools version 1.15.1 (*76*). Read group information was added to the combined bam file using AddOrReplaceReadGroups in Picard Tools version 2.23.9 (*77*), and exonic SNP positions were called using GATK version 3.6 (*78*) with “-stand_call_conf 50.0 -stand_emit_conf 10.0 –genotype_likelihoods_model SNP” parameters. Fixed differences between line A and line B were identified (data S1 to S3). The functional consequences of SNPs were annotated using SnpEff version 4.3 (*79*).

### Quantification of differential allelic expression and identification of candidate imprinted genes

The counts for the reference allele (line A allele) and the alternative allele (line B allele) for each called SNP were summarized and SNPs with low coverage (reference allele counts + alternative allele counts <6) in each tissue were discarded to retain informative SNPs. The alternative allelic expression ratio (AAER), defined as the count for the line B allele divided by the sum of the counts for line A and line B alleles, was calculated for the quantification of allelic expression in placenta (data S1), embryo (data S2), and maternal follicle (data S3). Distributions of allelic expressions (measured by AAER) were plotted to infer the maternal vs. embryonic origin of placental tissue (Fig. 1C).

To identify candidate imprinted genes, we focused on exonic SNPs exhibiting parent-of-origin dependent allelic expression. A commonly accepted criterion for imprinting was applied (*28*), requiring that >65% of allelic expression originate from the same parental allele in both reciprocal crosses. Because lines A and B are semi-inbred, we also needed to account for residual heterozygosity in these parental lines. To do so we applied another cut-off to include only informative SNPs between line A and line B. For SNPs with AAER <0.1 in P_A_ and AAER >0.9 in P_B_, those with AAER <0.35 in F_1_ and AAER >0.65 in F_1r_ were classified as candidate maternally biased SNPs, whereas SNPs with AAER >0.65 in F_1_ and AAER <0.35 in F_1r_ were classified as candidate paternally biased SNPs. Conversely, for SNPs with AAER >0.9 in P_A_ and AAER <0.1 in P_B_, those with AAER >0.65 in F_1_ and AAER <0.35 in F_1r_ were classified as candidate maternally biased SNPs, and SNPs with AAER <0.35 in F_1_ and AAER >0.65 in F_1r_ are considered as candidate paternally biased SNPs. Candidate maternally-biased SNPs are labeled in red in the top-left corner of Fig. 2A and candidate paternally-biased SNPs are labeled in blue in the bottom-right corner. Genes containing at least two independent SNPs (separated by a minimum distance equivalent to half the read length) that met our criteria for parent-of-origin expression were regarded as candidate maternally/paternally imprinted genes (candidate imprinted genes; data S4). We used Fisher’s exact test to identify SNPs with significant differential allelic expression between the two reciprocal crosses. Genes with at least two independent significant SNPs (adjusted *P* < 0.05) were classified as maternally/paternally expressed imprinted genes (MEGs and PEGs; data S4). Data visualization was performed using RStudio software (version 4.2.1).

### PacBio HiFi data processing and DNA methylation analysis

The PacBio HiFi BAM files of *P. prolifica* were aligned to the *P. prolifica* reference genome (GCF_027474105.1) using pbmm2. The alignment percentage was assessed using SAMtools (version 1.20). The methylated sites were identified using the aligned_bam_to_cpg_scores command implemented in pb-CpG-tools, which infers CpG methylation based on DNA polymerase kinetics recorded during PacBio sequencing. A total of 11,158,123 CpG sites with methylation information were identified. To ensure reliable methylation calling, only CpG sites with a minimum sequencing depth of 10× were retained. After filtering, 92.2% of CpG sites were retained with a mean coverage of 20×. For each CpG site, the numbers of methylated and unmethylated cytosines (Cs) were extracted for downstream analyses. The methylation level of each CpG site was calculated as the proportion of methylated Cs among all detected Cs at that site. Allele-specific DNA methylation was inferred by phasing long HiFi reads containing an informative heterozygous SNP and assigning CpG methylation calls to corresponding parental haplotypes.

### Validation of candidate imprinted genes by allele-specific pyrosequencing

Our ability to validate candidate imprinted genes was limited by the very small amounts of total RNA available from *P. prolifica* placenta due to the tiny size of the fish conceptus (see table S1). We therefore chose three genes for pyrosequencing validation that represent a range of parental allelic biases: paternally biased *dpp4* and maternally biased *clic2* and *smg9*. Primers for pyrosequencing were designed using PryoMark Assay Design Software Version 2.0.1.15 (Qiagen, CA, USA); sequences are listed in table S5. Biotinylated PCR products were prepared with PyroMark Q48 Advanced Reagents (Qiagen, CA, USA) using 20 ng cDNA input. For each sample, 10 μl of PCR product was added to the PyroMark Q48 Disc to start the run on the PyroMark Q48 instrument (Qiagen, CA, USA). Each assay had two technical replicates using two cDNA aliquots to ensure the accuracy of the results. *Dpp4* exhibited strong paternally-biased allelic expression based on both RNAseq data and pyrosequencing validation (See Main Text for details). Based on the RNAseq data, *smg9* was expressed exclusively from the line A maternal allele (F_1_ = 100%) but was only moderately maternally biased in the reciprocal cross (F_1r_ = 61%) (fig. S4C). Pyrosequencing identified a similar, albeit weaker, pattern of bias with 89% maternal expression in the F_1_ and 57% in the F_1r_ (fig. S4C). For *clic2*, maternal allelic bias in the RNAseq data was 67% in the F_1_ and 68% in the F_1r_ but was stronger in the F_1_ (95%) and weaker in the F_1r_ (48%) based on pyrosequencing (fig. S4F).

### Single-cell RNA sequencing data analysis

Raw single-cell RNA sequencing data from human placenta were downloaded from NCBI (GSE145036 and GSE173193) (*49*, *50*). The cellranger count pipeline (Cell Ranger, version 9.0.1) was used to align reads to the human genome (GRCh38, GCF_000001405.40) with the STAR aligner (version 2.7.2) (*75*). We used ‘cellranger aggr’ to generate normalized aggregate data from each dataset, and to produce the gene counts by cell matrix. We processed the count data using the R package Seurat (*80*). The cells with fewer than 100 genes or more than 10,000 genes and > 15% mitochondrial reads were removed. The processed data were merged and normalized using the ‘NormalizeData’, ‘FindVariableFeatures’, and ‘ScaleData’ commands. Principal component analysis (PCA) was performed with the ‘RunPCA’ function and batch effect was corrected with the R Harmony package (version 1.2.3) (*81*). The top 30 Harmony components were then visualized in two dimensions using a t-distributed stochastic neighbor embedding (t-SNE) plot. The SingleR package (version 2.6.0) (*82*) was employed to annotate cell types by calculating the Spearman coefficient between the single-cell gene expression profile and the reference dataset from ‘Human Primary Cell Atlas’ (*83*), retrieved using the HumanPrimaryCellAtlasData() function. The expression profiles of *DPP4* and other genes of interest were visualized using the ‘FeaturePlot’ function in Seurat.

### Gene neighborhood analyses

Gene neighborhood analyses were performed for three *P. prolifica* imprinted genes (*grb10b*, *slc38a2*, *dpp4*) using both the *P. prolifica* genome (GCF_027474105.1) (*73*) and the USCS genome browser (*84*). The genomic regions flanking each imprinted gene were examined in *P. prolifica* and in the human genome, and synteny analyses were visualized in Adobe Illustrator (version 2024).

### Identification of differentially expressed genes (DEGs) and functional enrichment analyses

Read counts for each BAM file were summarized using BEDTools (*85*). Genes with CPM (counts per million) >1 in at least six samples were retained. Raw read counts were normalized using the trimmed mean of M-values (TMM) method implemented in the edgeR package in R (version 3.6.4) (*86*). DEGs between placenta and embryo samples were identified using a cutoff of |log_2_(fold change)| > 2 and a false discovery rate (FDR) < 0.05 (data S5). Gene ontology (GO) term analyses were performed using ShinyGO (version 0.82) with an FDR cutoff of 0.05 (*87*).

### Evolutionary rate correlation analyses

Evolutionary rate correlation (*39*) was calculated using the basic method described in Varela *et al.* (2021) (*40*). The dataset was expanded to a set of 15,118 proteins across 71 mammalian species drawn from the NCBI ortholog database for phylogenetic diversity within mammals (*88*). The rate data were normalized to species rate distributions using IQR scaling (*89*), which provides an evolutionary rate for each protein on 39–71 phylogenetic branches (determined by the completeness of protein sequences per species). Spearman Rank correlations (*rho*) were then calculated for all protein combinations, weighted by sample size for the pair (n) using *rho* × sqr (n/71) (*90*). The top ERC partners for each protein were extracted from the matrix of all pairwise weighted Spearman rank correlations between the normalized rates of each pair of proteins.

The reciprocal ranks of the top ERC connections between proteins were calculated both for protein-to-partner and partner-to-protein. To identify proteins with strong reciprocal ranks, we required that both ranks fell below a threshold (e.g., 15). These represent the strongest candidates for protein-protein interactions. Networks were generated using the NetworkX package in Python (*91*). Enrichments were performed using the top one percent (151 out of 15,118) of ERC partners based on weighted *rho* values, and were calculated using ClusterProfiler run against the 2025-3-16 release of the GO database (*92*). This includes all 3 ontologies (Biological Pathway, Molecular Function, Cellular Component) restricted to annotation terms that contain 2–2000 proteins. The 15,118 orthologous proteins were used as the background set for the enrichments and *p*-values were FDR-adjusted using the R STATS package (*86*).

To compare the mammalian ERC data to *Poeciliopis prolifica*, the mammalian matrix was trimmed to the overlap orthology set using the gene annotations for *P. prolifica*. Orthologs were further augmented by identifying ortholog annotations between *P. prolifica* and zebrafish (*Danio rerio*), and converting those gene names to *Homo sapiens* using the babelgene package (*93*). This resulted in the 10,056 shared ortholog set (data S6). Protein paired ranks were recalculated for this trimmed set, which was used to evaluate gene networks present in mammals that include genes common to *P. prolifica*. For the top 5 proteins identified in the DPP4 network, an enrichment analysis was performed against the background of shared orthologs (data S7).
