# Supplementary material for: Independent origin of genomic imprinting in placental fish
Source: Sci Adv. 2026 Nov 4;12(45):eaeh5487. doi: 10.1126/sciadv.aeh5487 (PMC13733801; doi:10.1126/sciadv.aeh5487)
Supplement: Supplementary file 1 [file sciadv.aeh5487_sm.pdf]

Science Advances
AAAS

Supplementary Materials for

Independent origin of genomic imprinting in placental fish

Ying Zhang *et al.*

Corresponding author: David Reznick, david.reznick@ucr.edu; Xu Wang, xzw0070@auburn.edu

Sci. Adv. **12**, eaeh5487 (2026)
DOI: 10.1126/sciadv.aeh5487

This PDF file includes:

Figs. S1 to S10
Tables S1 to S5
Legends for data S1 to S7

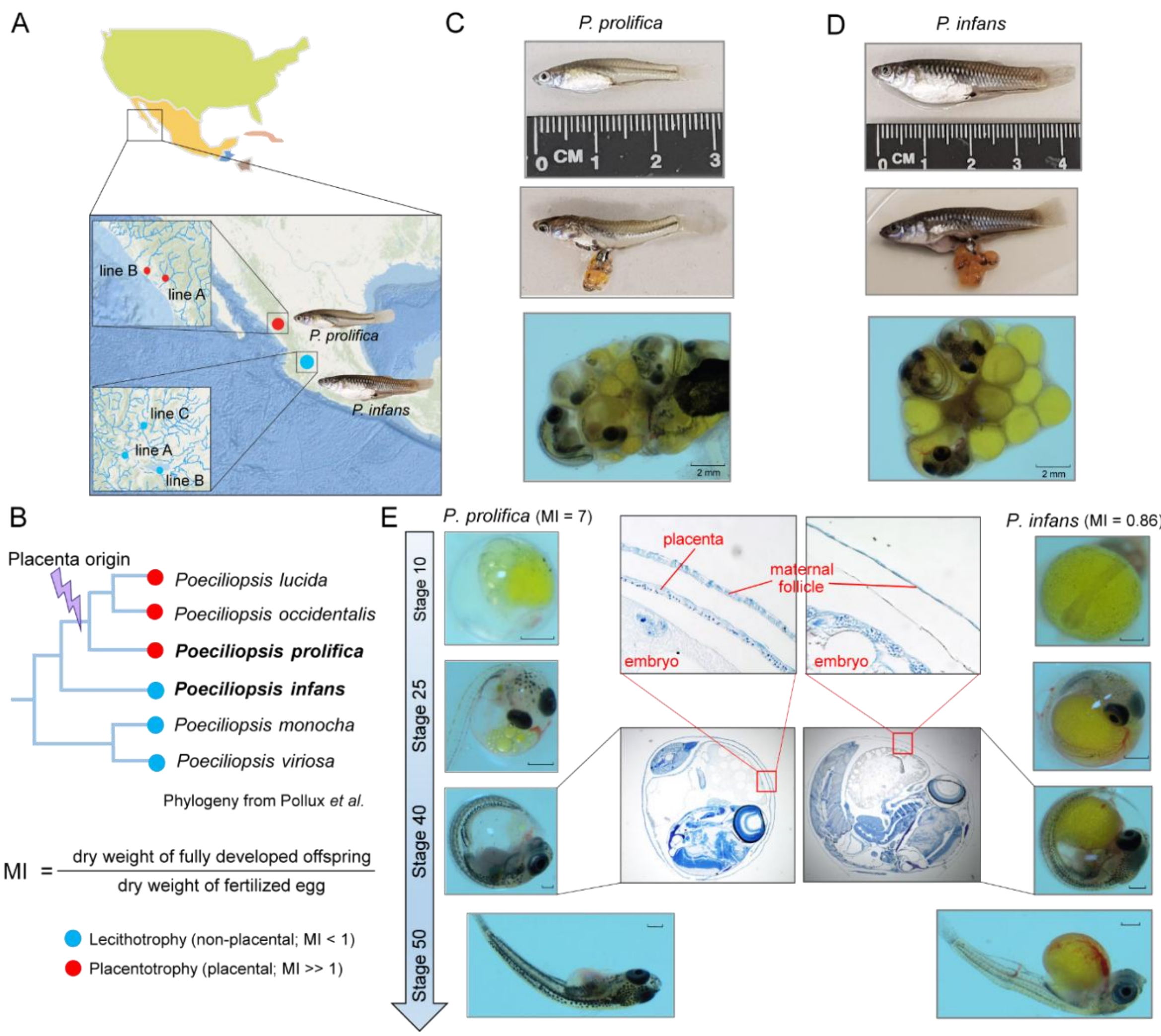

fig. S1. Comparative phylogeny and reproductive biology of placental species *Poeciliopsis prolifica* and non-placental species *P. infans.*

(A) Geographical distribution of the two focal species, showing the map for north and central America (***upper panel***) and waterway map of collection sites (***lower panel***) for different lines of *P. prolifica* (red circles) and *P. infans* (blue circles). **(B)** Phylogenetic tree showing species with different modes of reproduction in the genus *Poeciliopsis*. Blue circles represent lecithotrophic non-placental species, red circles represent matrotrophic placental species. *P. prolifica* and *P. infans* are highlighted in bold. **(C** and **D)** Morphology and female reproductive structure for *P. prolifica* (**C**) and *P. infans* (**D**). From ***top*** to ***bottom***: pregnant female fish, the same female after ovary dissection, and an entire brood imaged under a stereomicroscope. **(E)** Ontogeny of embryonic and placental structures. Representative embryos at developmental stages 10 (uneyed), 25 (mid-eyed), 40 (late-eyed) and 50 (very late-eyed) are shown for each species (staging after (*84*)). The middle panels depict hematoxylin-and-eosin sections through the embryonic surface and placental complex at stage 40, demonstrating placental tissues in *P. prolifica* (matrotrophy index, MI = 7.0) and a thin chorionic interface in *P. infans* (MI = 0.86). The length of the scale bar is 500 µm. Portions of this figure (the embryo photograph and simplified geographic map) are reproduced in Fig. 1 to provide context for the main text.

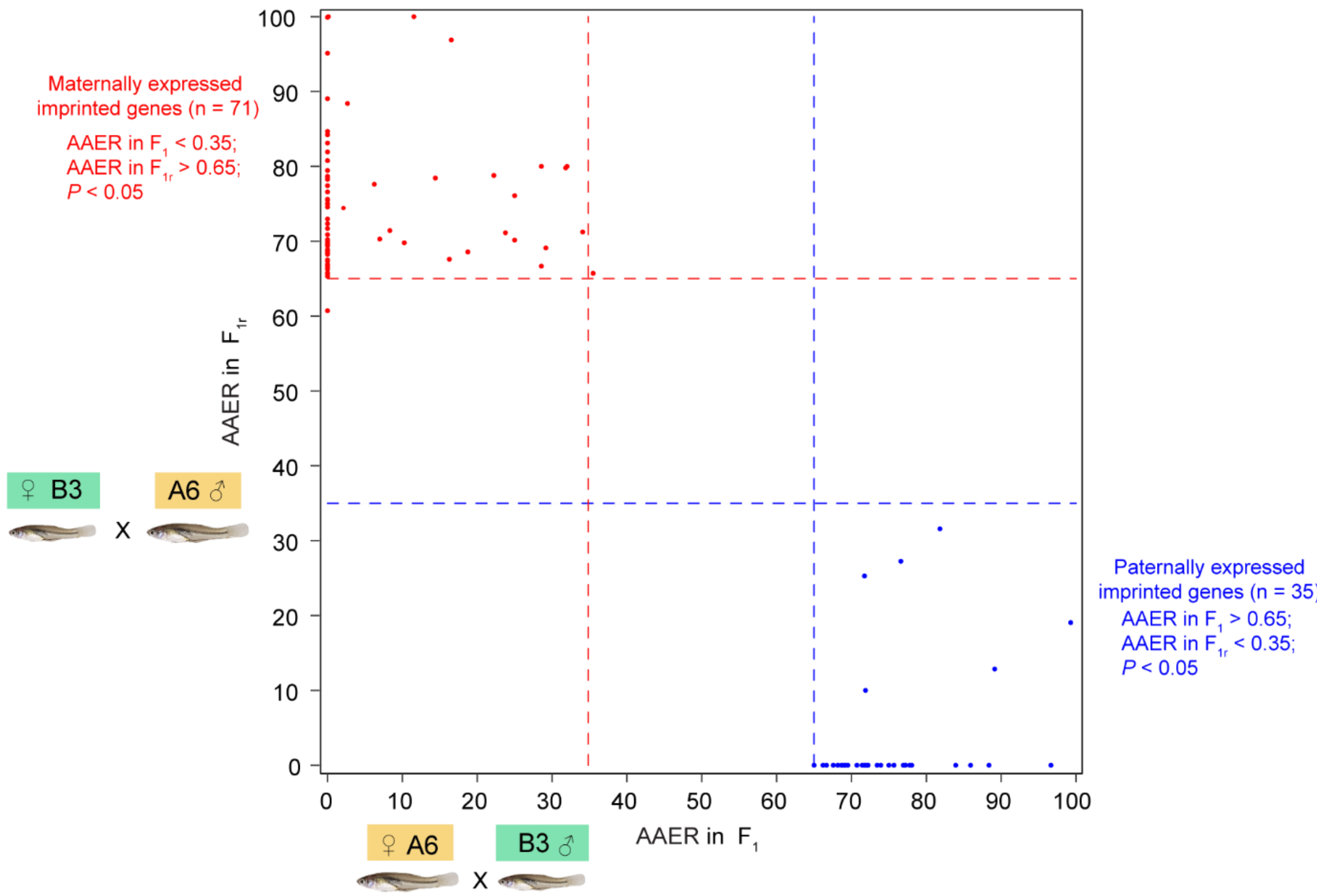

fig. S2. Scatterplot of allelic expression of imprinted genes identified in *Poeciliopsis prolifica* placenta.

Each point in the scatterplot represents a high-confidence imprinted gene ($P < 0.05$, Fisher's exact test) identified in placental transcriptomes from the two reciprocal crosses: ♀ A6 × ♂ B3 (F_1; horizontal axis) and ♀ B3 × ♂ A6 (F_{1r}; vertical axis). The Alternative Allele Expression Ratio (AAER) for each gene is calculated as the sum of allelic expression from line B, divided by the sum of allelic expression from line A plus line B. Maternally expressed imprinted genes are represented using red dots (upper-left dashed box), with selection criteria of AAER < 0.35 in F_1 and AAER > 0.65 in F_{1r}. Paternally expressed imprinted genes are represented using blue dots (bottom-right dashed box), with selection criteria of AAER > 0.65 in F_1 and AAER < 0.35 in F_{1r}.

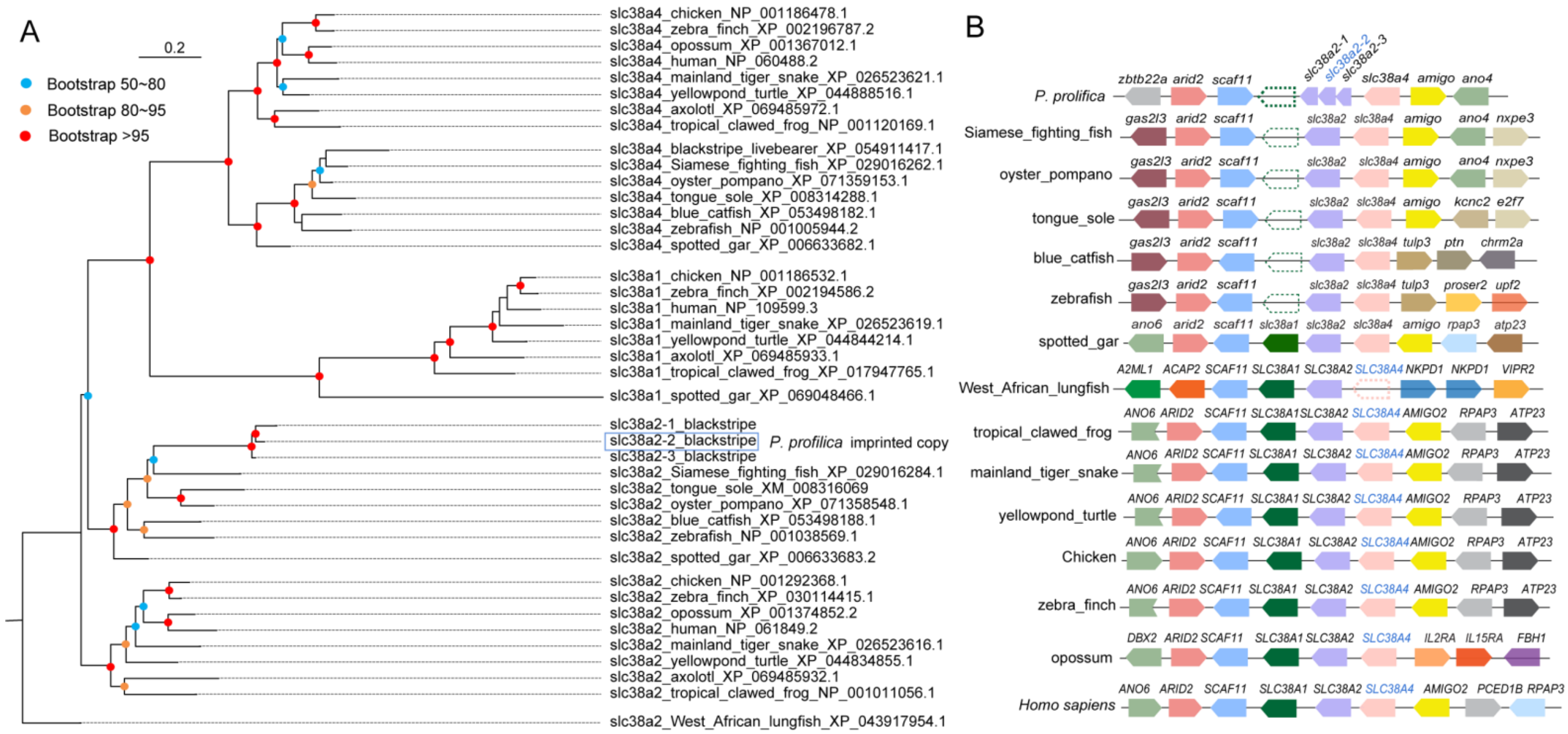

fig. S3. Evolutionary relationships and conserved synteny of the *SLC38A* gene family across vertebrates.

(A) Maximum likelihood tree of protein sequences from *SLC38A* family (A1, A2, and A4), using their orthologs in West African lungfish as the outgroup. Nodes with bootstrap values are indicated by colored circles (blue, 50~80%; orange, 80~95%; red, >95%). (**B**) Conserved gene synteny in the genome neighborhood is shown for each species. The gene synteny in the middle panel, and dotted line boxes represent the loss of *slc38a1* in teleost species after WGD and the loss of *slc38a4* in the West African lungfish.

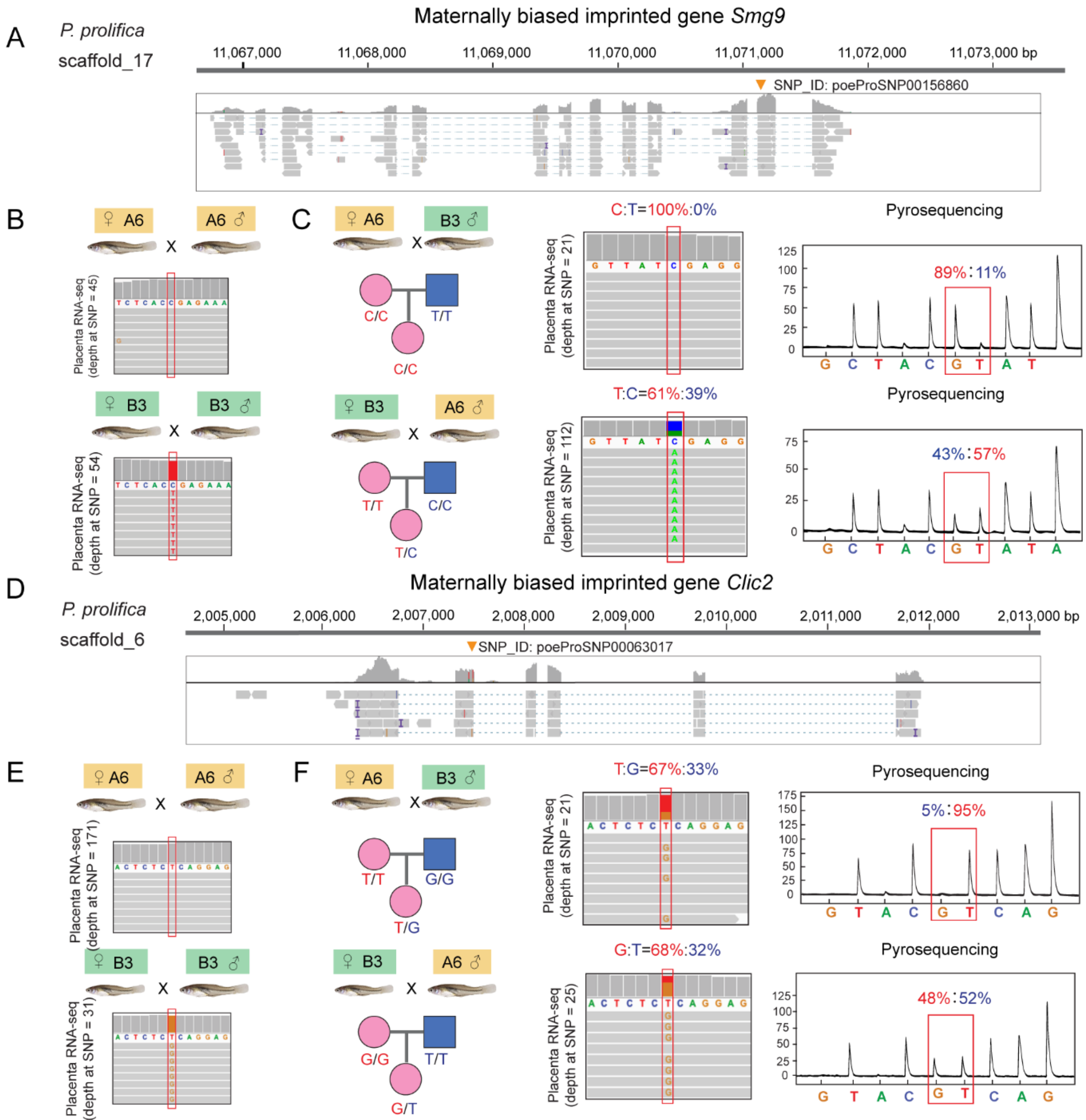

Fig. S4. Pyrosequencing validation for maternally biased candidate imprinted genes *smg9* and *clic2* in *Poeciliopsis prolifica* placenta.

(A to **C)** Validation of the *smg9* locus. **(A)** Integrative Genomics Viewer (IGV) pile-up of placental RNA-seq reads aligned to *smg9* gene region. Genomic location of informative exonic SNP is indicated with orange arrow. **(B)** RNA-seq alignments in the semi-inbred parental crosses. **(C)** Pyrosequencing validation in reciprocal F_1 crosses. ***Left**:* pedigrees illustrating the cross design (paternal allele is labeled in blue and maternal is labeled in pink). ***Middle***: differential allelic expression inferred from RNA-seq data. ***Right***: pyrosequencing validation DAE showing the pyrograms. **(D** to **F)** Validation of the *clic2* locus. **(D)** IGV pile-up of placental RNA-seq reads aligned to *clic2* gene region. **(E)** RNA-seq alignments in the semi-inbred parental crosses. **(F)** Pyrosequencing validation in reciprocal F_1 crosses.

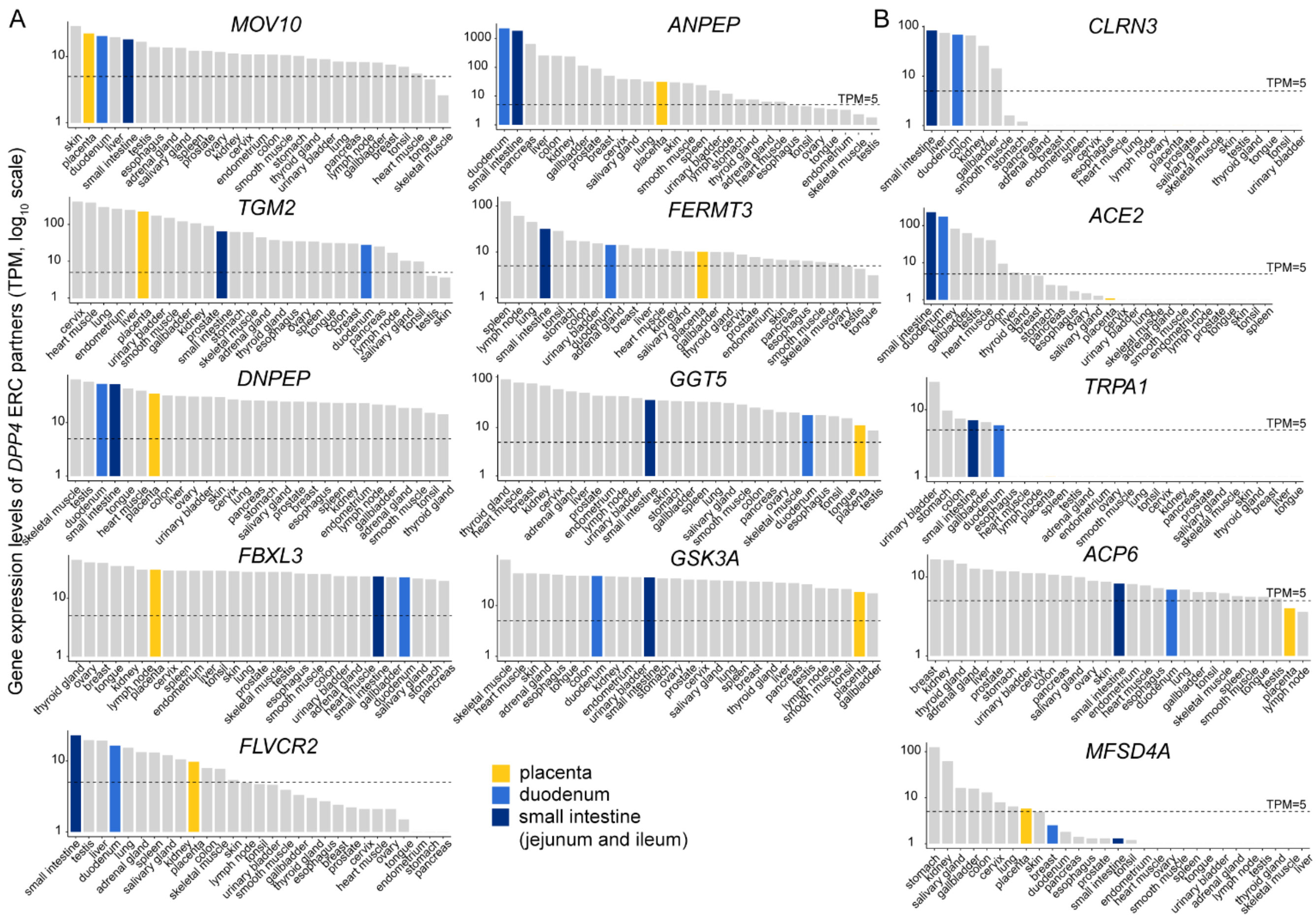

fig. S5. Expression levels of *DPP4* top 15 ERC partners in human tissues.

Barplots of $\log_{10}$ transformed Transcripts Per Million (TPM) values for *DPP4* top 15 ERC partners across human tissues (see Fig.5a and Methods). **(A)** Genes ($n = 9$) with TPM > 5 in both the placenta (yellow) and the intestine (blue). In the Human Protein Atlas, duodenum is categorized as a distinct tissue (shown in light blue) and the small intestine primarily represents jejunum and ileum (shown in dark blue). The dashed line indicates TPM = 5. **(B)** Genes ($n = 5$) with TPM > 5 in either the placenta (yellow) or the duodenum/small intestine (light blue/dark blue). The dashed line indicates TPM = 5.

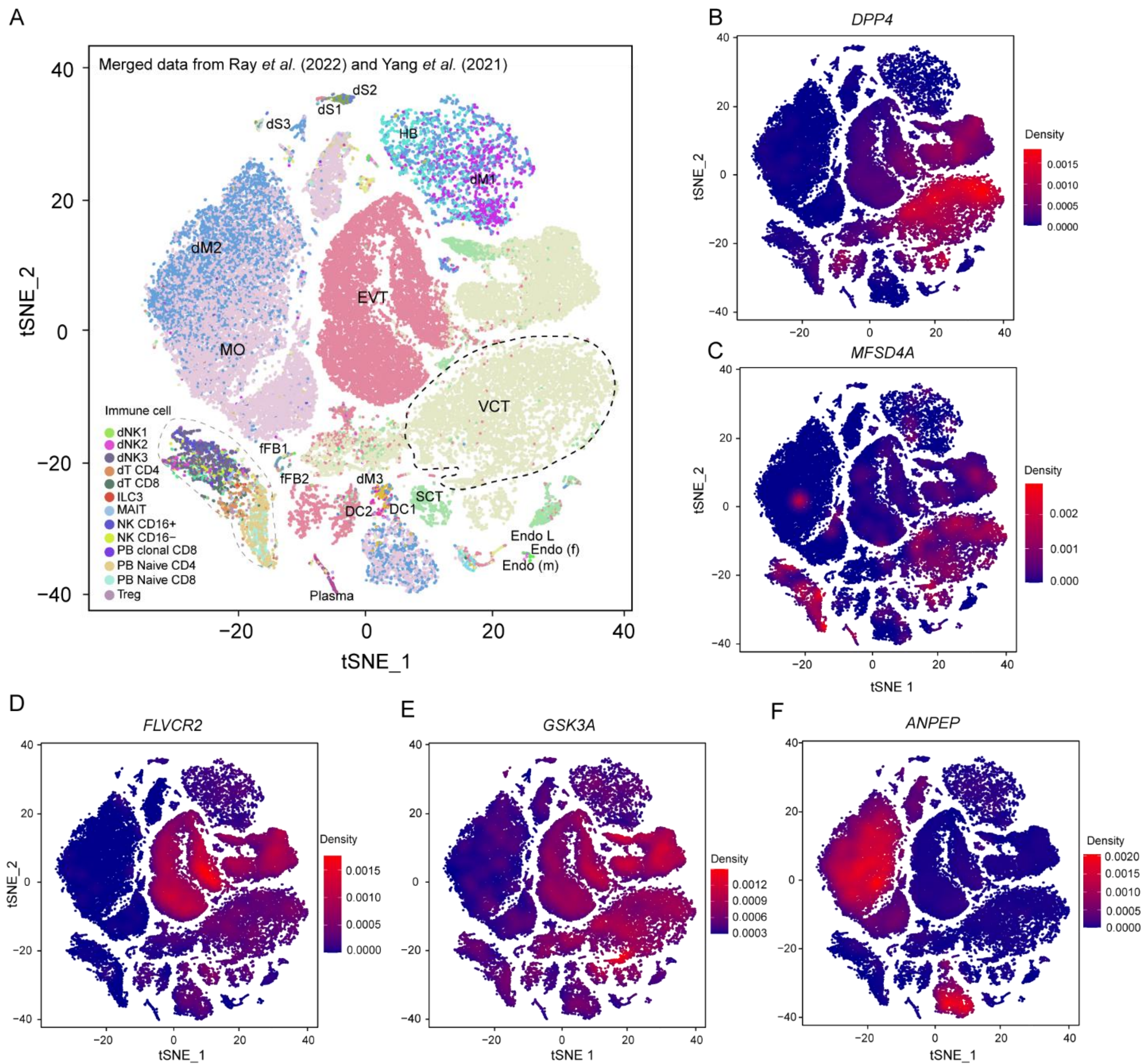

fig. S6. *DPP4* and its ERC partners expression landscape in human placenta revealed by single-cell RNA-sequencing.

(A) T-distributed stochastic neighbor embedding (tSNE) plot of cell types identified in the human placenta. Data are merged from the studies of Ray *et al*. (2022) (*41*) and Yang *et al*. (2021) (*42*). The dashed ellipse highlights the cell type with the highest *DPP4* expression. d, decidual; DC, dendritic cells; dM, decidual macrophages; dS, decidual stromal cells; NK, natural killer cell; Treg, regulatory T cell; MO, monocytes; MAIT, mucosal-associated invariant T cell; HB, Hofbauer cells; PB, peripheral blood; EVT, extravillous trophoblast; VCT, villous cytotrophoblast; SCT, syncytiotrophoblast; FB, fibroblasts; Endo, endothelial cells. **(B** to **F)** Expression density of *DPP4 (***B***)* and its ERC partners *MFSD4A* (**C**), *FLVCR2* (**D**), *GSK3A* (**E**), and *ANPEP* (**F**).

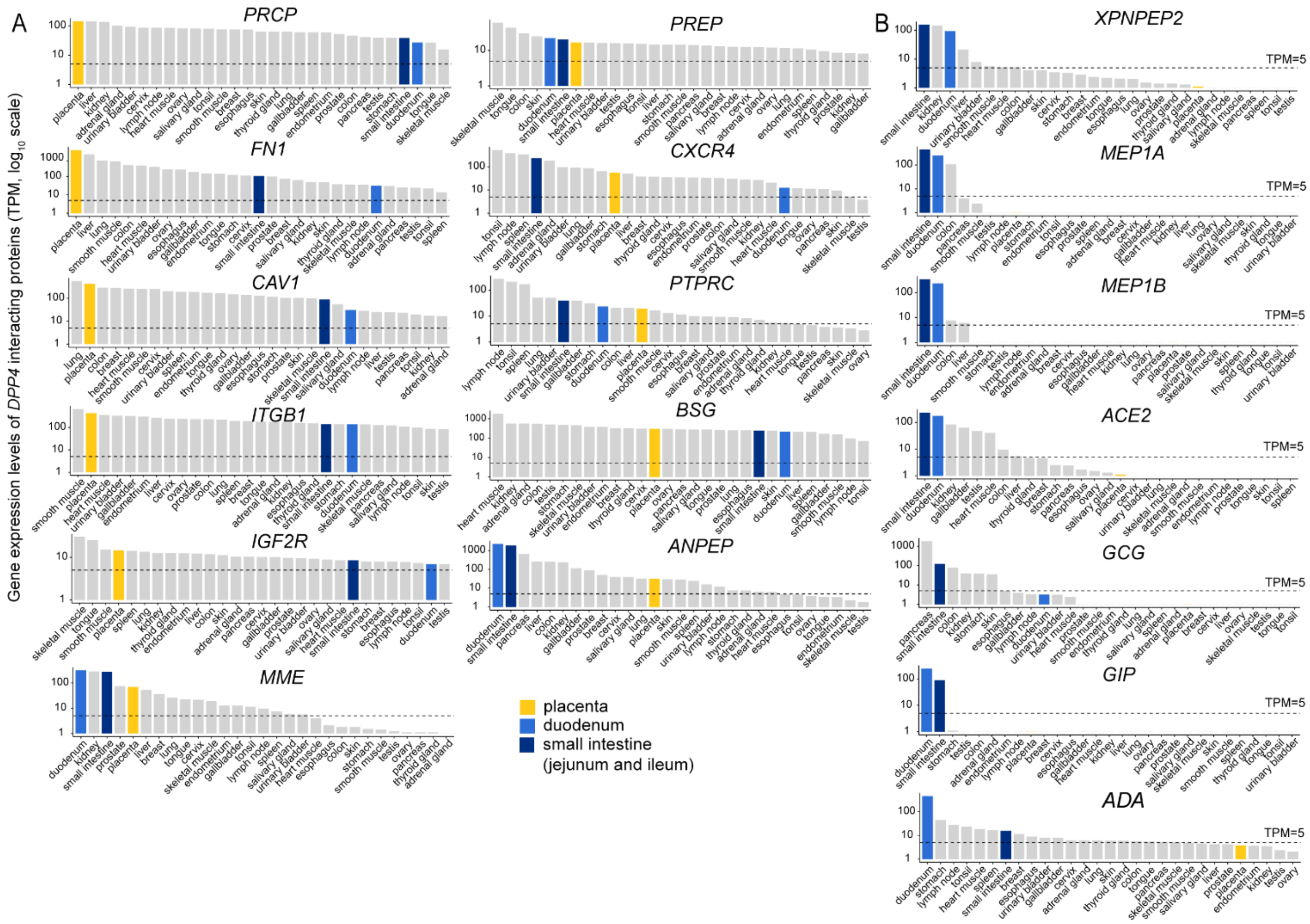

fig. S7. Expression levels of *DPP4*-interacting proteins in human tissues.

Barplots of $\log_{10}$ transformed Transcripts Per Million (TPM) values for *DPP4*-interacting proteins across human tissues (data from *human protein atlas*; see Fig.6A and Methods). (**A**) Genes with TPM > 5 in both the placenta (yellow) and the intestine (blue). In the Human Protein Atlas, duodenum is categorized as a distinct tissue (shown in light blue) and the small intestine primarily represents jejunum and ileum (shown in dark blue). The dashed line indicates TPM = 5. **(B)** Genes (n = 5) with TPM > 5 in either the placenta (yellow) or the duodenum/small intestine (light blue/dark blue).

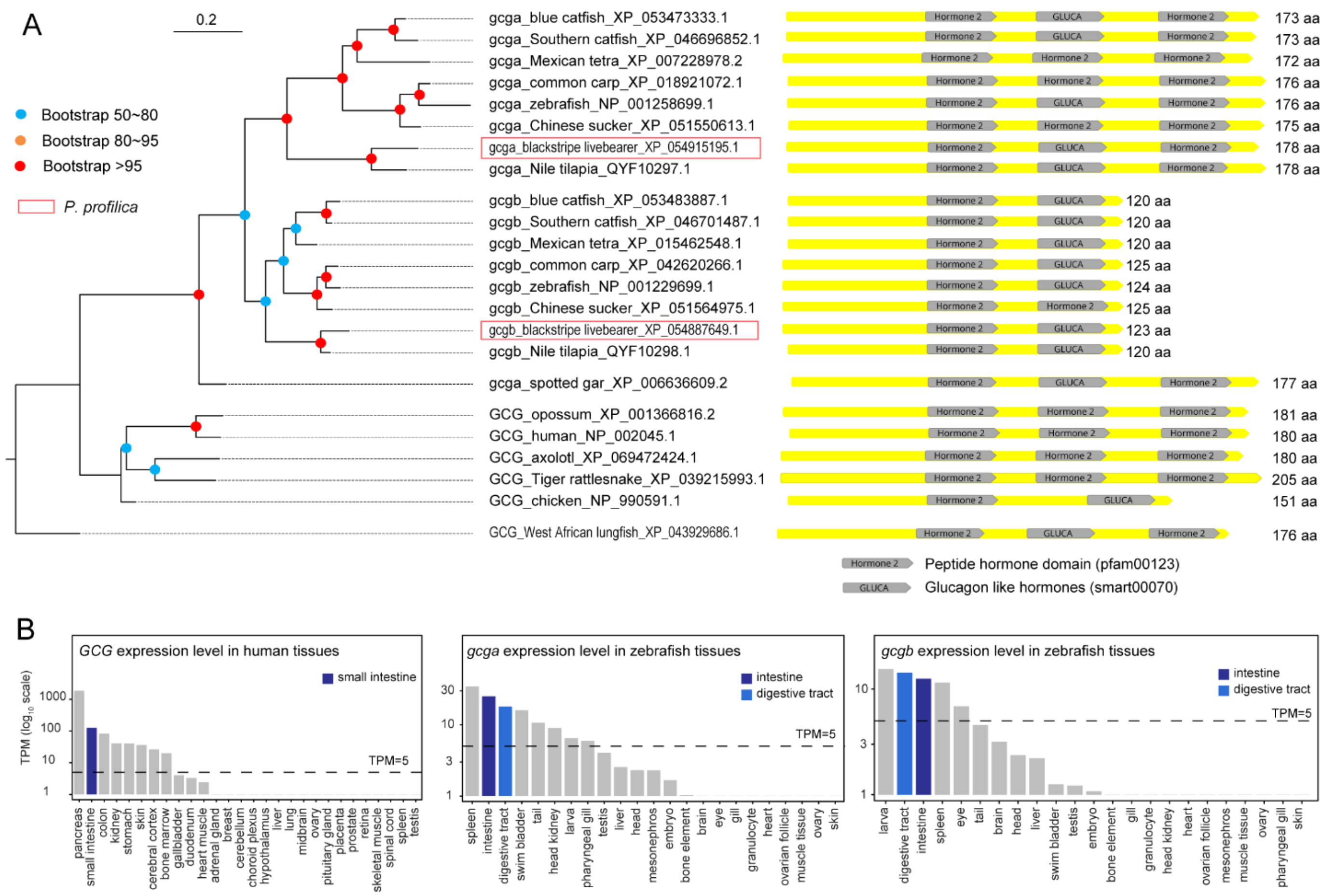

fig. S8. Evolutionary relationships, domain protein architecture, and tissue-specific expression of GCG across vertebrate species.

(**A**) Maximum-likelihood phylogenetic analysis of vertebrate *GCG* proteins. Branch support values are indicated by colored circles (blue, 50~80%; orange, 80~95%; red, >95%). The tree identifies three major clades corresponding to the two ohnologs (*gcga* and *gcgb*), and vertebrate *GCG* orthologs. Protein length (number of amino acids, aa) and domain architecture are shown in schematic diagrams to the right. The Hormone 2 (pfam00123) and Glucagon-like hormone (smart00070) domains are demonstrated in gray boxes. *P. prolifica* gcga protein sequence downloaded from NCBI (XP_054915195.1) is 217 aa in length, and it is trimmed to 178 aa based on the transcript models. (**B**) Tissue-specific expression profiles of *GCG* genes based on publicly available transcriptomic datasets. ***Left***, human *GCG* expression is highly enriched in the small intestine and pancreas (data from Human Protein Atlas). ***Middle***, zebrafish *gcga* is predominantly expressed in the spleen, intestine and digestive tract. ***Right***, zebrafish *gcgb* is primarily expressed in larva, the intestine and digestive tract, with lower expression detected in additional tissues (data from https://www.bgee.org). Expression values are shown as TPM on a $\log_{10}$ scale. Colored bars highlight the small intestine and digestive tract tissues. The dashed line represents the threshold for tissues with detectable expression (TPM = 5).

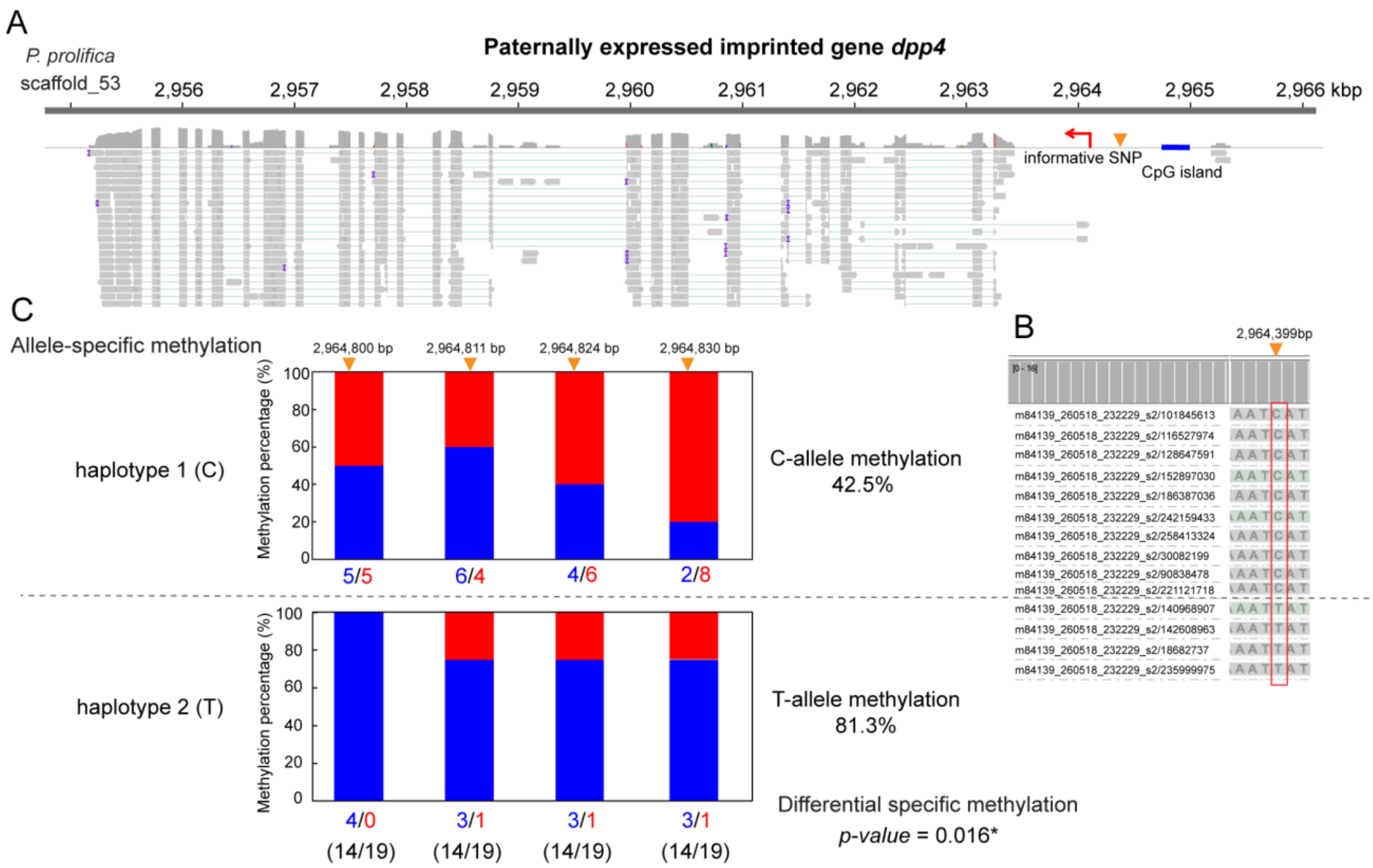

fig. S9. Allele-specific methylation quantification in the promotion CpG island of the paternally expressed imprinted gene *dpp4*.

(A) Integrative Genomics Viewer (IGV) pile-up of *Poeciliopsis Prolifica* A6×B3 placenta RNA-seq reads aligned to the *dpp4* gene region, showing the exon structures. An informative heterozygous SNP (orange arrow; position 2,964,399) located adjacent to a CpG island (blue bar) enabled haplotype-resolved methylation analysis. **(B)** Alignment of PacBio HiFi reads spanning the informative SNP position 2,964,399 bp, showing the two alleles (C and T) used to assign reads to the corresponding parental haplotypes. The sequencing coverage at the CpG island is 19. Of these, only 14 reads could be assigned to a haplotype based on the corresponding informative SNP, with 10 reads carrying the C allele and 4 reads carrying the T allele at each locus. **(C)** Allele-specific DNA methylation levels at 4 CpG sites within the CpG island. Blue and red bars represent methylated and unmethylated cytosines, respectively. Numbers beneath each bar indicate the counts of methylated and unmethylated reads at each CpG site. Overall methylation across these 4 CpGs was significantly higher for the T allele (81.3%) than for the C allele (42.5%; $P = 0.016$, Fisher's exact test).

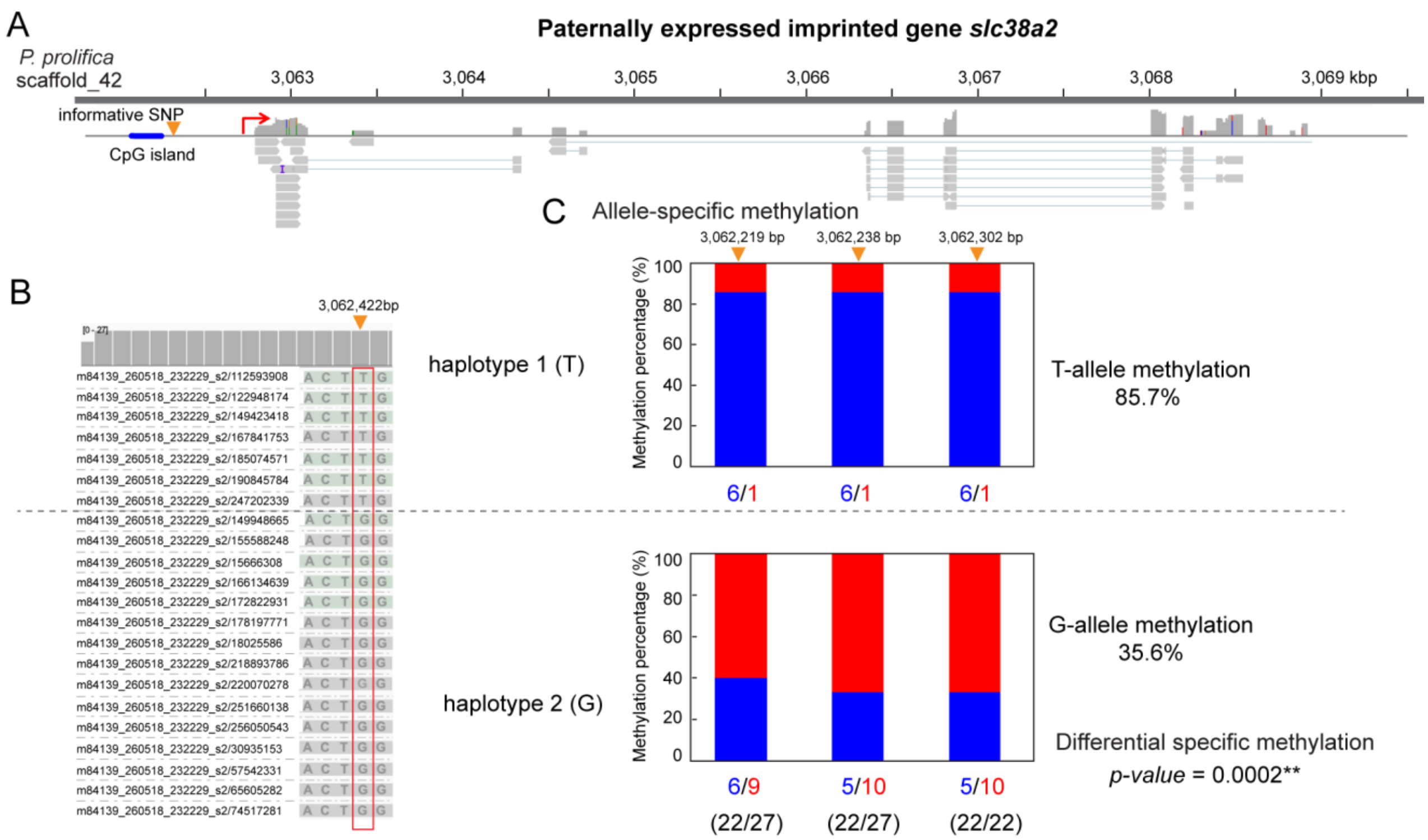

fig. S10. Allele-specific methylation quantification in the promotion CpG island of the paternally expressed imprinted gene *slc38a2*.

(A) Integrative Genomics Viewer (IGV) pile-up of *Poeciliopsis Prolifica* A6×B3 placenta RNA-seq reads aligned to the *slc38a2* gene region, showing the exon structures. An informative heterozygous SNP (orange arrow; position 3,062,422) located adjacent to a CpG island (blue bar) enabled haplotype-resolved methylation analysis. **(B)** Alignment of PacBio HiFi reads spanning the informative SNP position 3,062,422 bp, showing the two alleles (T and G) used to reads to the corresponding parental haplotypes. The sequencing coverage at the CpG island locus is 27. Of these, only 22 reads could be assigned to a haplotype based on the corresponding informative SNP, with 7 reads carrying the T allele and 15 reads carrying the G allele at each locus. **(C)** Allele-specific DNA methylation levels at 3 CpG sites within the CpG island. Blue and red bars represent methylated and unmethylated cytosines, respectively. Numbers beneath each bar indicate the counts of methylated and unmethylated reads at each CpG site. Overall methylation across these 3 CpGs was significantly higher for the T allele (85.7%) than for the G allele (35.6%; $P = 0.002$, Fisher's exact test).

table S1. Summary of RNA-seq yield and genome mapping rate of embryonic, placental and maternal follicle tissues from *Poeciliopsis prolifica* A and B lines.

Sample	Tissue	RNA conc. (ng/μL)	Raw reads	Filtered reads	Mapped reads	STAR%	TopHat %
poeProA6xA6_stage35	Embryo1	25.3	37,695,114	35,772,720	19,835,405	52.94%	55.45%
poeProA6xA6 _stage45	Embryo2	92.2	56,173,926	52,623,496	41,437,302	83.88%	78.74%
poeProA6xA6 _stage40	Embryo7	89.6	48,613,472	45,485,002	38,609,293	88.49%	84.88%
poeProA6xA6 _stage45	Follicle2	40.8	58,202,776	53,307,686	44,534,789	61.15%	83.54%
poeProA6xA6 _stage45	Placenta2	23.6	53,139,086	50,254,872	36,529,926	58.96%	72.69%
poeProA6xA6 _stage40	Placenta7	10.7	39,327,160	36,710,370	21,139,265	58.80%	57.58%
poeProB3xB3 _stage40	Embryo4	324	105,723,702	97,624,496	79,207,924	89.17%	81.14%
poeProB3xB3 _stage40	Embryo11	71.6	52,052,272	48,989,732	40,677,191	91.86%	83.03%
poeProB3xB3 _stage40	Follicle4	30.4	52,415,246	49,003,444	24,426,137	38.95%	49.85%
poeProB3xB3 _stage40	Placenta4	15.1	53,034,868	49,856,614	24,467,402	40.64%	49.08%
poeProB3xB3 _stage40	Placenta11	11.4	46,675,958	43,834,692	37,438,915	91.19%	85.41%
poeProA6xB3 _stage40	Embryo1	96.8	94,602,604	87,120,706	62,518,787	50.42%	71.76%
poeProA6xB3 _stage45	Embryo5	100	49,961,458	47,021,976	38,429,285	90.02%	81.73%
poeProA6xB3 _stage45	Embryo8	87.6	82,237,462	75,383,700	53,532,373	60.19%	71.01%
poeProA6xB3 _stage45	Follicle8	33.4	90,841,614	78,633,266	39,960,393	37.64%	50.82%
poeProA6xB3 _stage40	Placenta1	24.4	79,421,500	72,677,146	53,326,943	60.61%	73.38%
poeProA6xB3 _stage45	Placenta5	11.9	48,744,514	46,029,668	39,537,060	63.98%	85.89%
poeProB3xA6 _stage40	Embryo1	340	257,368,608	246,387,218	204,654,608	87.60%	83.06%
poeProB3xA6 _stage40	Embryo2	87.6	112,756,648	103,207,952	77,597,086	77.61%	75.19%
poeProB3xA6 _stage40	Embryo9	83	56,421,966	52,775,892	44,150,931	90.49%	83.66%
poeProB3xA6 _stage40	Follicle1	31.6	97,474,252	89,939,558	69,963,458	77.82%	77.79%
poeProB3xA6 _stage40	Follicle2	34.6	135,478,944	124,167,148	89,953,971	76.89%	72.45%
poeProB3xA6 _stage40	Placenta1	10	107,488,140	100,985,426	63,633,401	67.04%	63.01%
poeProB3xA6 _stage40	Placenta2	41.6	100,714,272	91,677,802	66,713,051	73.96%	72.77%
poeProB3xA6 _stage40	Placenta9	21.6	55,790,824	52,136,812	42,064,427	85.94%	80.68%

table S2. SNP-aggregated maternal versus paternal allelic counts in reciprocal F1 hybrids for maternally expressed protein-coding imprinted genes.

Gene name	Number of SNPs	Sum of MEA in F_1	Sum of PEA in F_1	Sum of MEA in F_{1r}	Sum of PEA in F_{1r}	*P*_Values
abhd5	8	198	0	391	178	6.5186E-78
agla	2	131	0	44	22	9.709E-28
c19orf12	2	35	0	36	12	1.7301E-13
cdab	3	38	0	73	33	2.0228E-15
cds2	2	13	3	24	11	0.00195024
clic2	2	38	19	51	21	3.51E-05
copb2	3	29	0	31	10	1.6278E-11
cxorf38	2	15	5	35	11	0.00022509
dhx30	2	43	0	64	13	2.4781E-21
dsg2l	2	31	0	646	117	1.4861E-24
eif2b5	3	67	0	57	25	1.2303E-21
eprs1	2	65	0	24	11	5.23E-15
fam136a	2	27	0	59	16	7.451E-14
fubp3	2	68	0	39	13	1.1225E-20
glrx	2	260	0	404	110	2.543E-117
gorasp1a	2	25	0	33	11	1.4811E-10
grb10b	2	21	0	69	32	1.5507E-09
hnrnpl	2	30	2	52	15	5.1681E-12
ibtk	2	44	0	76	30	2.502E-18
itm2c	2	31	0	50	23	3.9842E-12
kctd7	2	2	92	53	247	9.6244E-49
krt18	2	49	0	24	8	4.5623E-14
lyrm5a	2	17	0	31	15	8.2026E-07
man2b1	2	14	4	26	7	0.00021567
mao	2	524	0	275	146	4.671E-130
mpdu1b	2	18	6	47	20	0.00020628
msna	5	182	5	221	29	1.3214E-83
nop53	2	87	0	107	56	4.2356E-29
nop56	5	83	0	119	52	3.727E-31
nt5c2l1	2	24	0	37	12	1.0568E-10
pa2g4a	2	35	14	28	7	5.9544E-06
plpp2a	3	93	29	170	69	6.9371E-18
ppil4	2	34	14	85	38	2.5635E-06
ppp4cb	5	60	0	96	28	4.1119E-27
rab2a	4	169	0	657	279	5.1833E-77
rab7a	2	44	0	21	5	1.7066E-13
retsat	3	100	0	99	42	3.8054E-34
rhbdd1	3	32	0	42	19	7.2483E-12
rims2	2	17	8	16	4	0.00234282
rpe65b	2	23	0	48	25	7.2151E-09
rundc3aa	5	109	0	355	121	1.0707E-53
scn4a	3	43	0	64	12	8.9651E-22
sgf29	5	91	0	455	56	3.6033E-69
shroom1	6	131	0	193	59	2.7127E-56
siae	2	72	0	46	23	3.6023E-20
slc25a35	4	46	1	230	79	6.6693E-23
slc25a36b	2	20	0	27	13	1.5442E-07
slc30a4	2	28	0	79	40	9.8101E-12
smad2	3	56	0	58	15	6.0972E-23
smg9	2	43	0	112	72	2.05E-15
sox4b	2	28	0	68	26	6.3028E-13
supt5h	2	22	2	60	24	2.3967E-08
thap4	2	37	0	1305	67	1.4067E-45
tmem141	2	99	0	43	20	3.6943E-24
zadh2	2	178	0	178	88	7.5203E-57

MEA: Maternally expressed allele; PEA: Paternally expressed allele.

table S3. SNP-aggregated maternal versus paternal allelic counts in reciprocal F1 hybrids for paternally expressed protein-coding imprinted genes.

Gene name	Number of SNPs	Sum of MEA in F1	Sum of PEA in F1	Sum of MEA in F1r	Sum of PEA in F1r	*P*_Values
alg10	2	15	38	0	247	2.7976E-36
amot	2	15	30	0	246	5.2101E-30
ankrd13d	2	4	10	0	16	3.33E-05
apex1	2	12	29	0	134	7.2977E-24
atp13a2	2	18	45	0	40	1.3602E-14
crebzf	2	25	57	0	468	3.9036E-58
dpp4	2	42	0	115	46	3.9363E-19
f8	2	10	22	0	183	1.0709E-22
ier2a	2	24	47	0	956	1.1411E-63
krt5	3	5	144	0	2390	1.06E-230
morc2	2	24	50	0	232	1.9122E-39
napsa	2	20	122	0	208	1.3205E-73
nudcd2	2	9	28	0	66	9.5022E-18
nxpe3	3	24	62	0	330	1.9371E-54
pmf1	3	21	46	0	625	7.4646E-56
podxl	2	14	26	0	76	4.098E-16
rhoa-c	2	9	23	0	29	7.5983E-10
slc2a1a	3	9	47	0	596	5.758E-63
slc38a2	2	5	41	23	156	6.165E-23
smyd2a	2	9	23	6	54	2.0623E-09
srsf1	2	13	33	23	68	3.3382E-07
tbc1d16	2	9	32	0	135	2.5854E-27
tfb2m	2	8	27	0	71	2.0001E-18
tnfrsf14l	4	38	105	0	111	2.321E-39
tpcn1	2	13	29	0	408	6.4656E-36
tubgcp4	2	10	26	0	149	7.3273E-24
uvrag	3	447	113	535	0	1.125E-199
zfpm1	2	37	83	0	930	2.3651E-94
znf711	2	6	20	0	160	6.5771E-22

MEA: Maternally expressed allele; PEA: Paternally expressed allele.

table S4. Significant placenta-embryo differential expression of imprinted protein-coding genes.

**Gene_name**	**Bias**	**LogFC**	**LogCPM**	***P*-Value**	**FDR**	**Regulation**
dpp4	paternal	4.67441	6.86234	2.9889E-15	3.1374E-13	up
slc2a1a	paternal	-6.57956	4.31993	3.3509E-13	2.3872E-11	down
napsa	paternal	2.68622	6.75051	9.6425E-07	1.1951E-05	up
podxl	paternal	3.92723	6.81960	1.0995E-05	9.7146E-05	up
rims2	maternal	-3.83250	5.96727	1.0715E-13	8.4734E-12	down
retsat	maternal	2.86884	5.11378	8.0565E-11	3.0777E-09	up
itm2c	maternal	-3.06751	5.80126	1.091E-08	2.3899E-07	down
plpp2a	maternal	2.45701	4.87137	1.3385E-08	2.853E-07	up
kctd7	maternal	2.39893	7.30862	2.9754E-07	4.3145E-06	up
slc25a35	maternal	3.37869	4.34301	4.5165E-07	6.1613E-06	up
thap4	maternal	3.83460	4.84624	7.0735E-07	9.0755E-06	up
cdab	maternal	3.95856	4.68229	9.2403E-07	1.1513E-05	up
nt5c2l1	maternal	3.92262	5.71922	1.3341E-06	1.5856E-05	up
mao	maternal	2.58689	5.60838	3.8336E-06	3.9506E-05	up
siae	maternal	2.06092	3.28619	8.4238E-06	7.7247E-05	up
man2b1	maternal	2.00959	4.02068	8.894E-05	0.00058658	up
rundc3aa	maternal	-2.06457	6.51402	0.00155203	0.00671924	down
rpe65b	maternal	-2.47261	5.33874	0.00312266	0.01215204	down

table S5. Allele-specific pyrosequencing primer sequences used to validate parentally biased gene expression.

Target gene	Imprinting status	Primer orientation	Primer sequences (5'-3')	Tm (°C)
dpp4	paternally biased	forward	ACTTCAGCCACAATGCTTCTTAC	52.8
		biotin-reverse	CATCGCTAAATACCTTGTTGTCC	
		pyro	TCCCAGAATTCCACAG	
clic2	maternally biased	forward	TGCAGAGTTGTGGTCGATTTC	52.8
		biotin-reverse	CATCAAGAATAGCCCAAACAATG	
		pyro	CAAACGGCGAAACTC	
smg9	maternally biased	forward	GTGGAAAAGGTGGGGTGAC	52.8
		biotin-reverse	GCTTCCGGTCCAAGAAAATG	
		pyro	AAAAGGTGGGGTGAC	

Data S1 to S7 included in Dryad: https://doi.org/10.5061/dryad.ns1rn8q8r

data S1. Single-nucleotide polymorphisms (SNPs) and allelic expression counts in the placental samples of *Poeciliopsis prolifica*.

data S2. Single-nucleotide polymorphisms (SNPs) and allelic expression counts in the embryo samples of *Poeciliopsis prolifica*.

data S3. Single-nucleotide polymorphisms (SNPs) and allelic expression counts in the maternal follicle samples of *Poeciliopsis prolifica*.

data S4. Paternally and maternally expressed candidate imprinted genes in the placenta of *Poeciliopsis prolifica*.

data S5. Significantly differentially expressed genes (DEGs) identified between the placenta and embryo of *Poeciliopsis prolifica*.

data S6. A list of shared orthologous genes between *Poeciliopsis prolifica* and humans used as the background set used in the evolutionary rate correlation (ERC) analysis.

data S7. Significantly enriched pathways among the top 5 *dpp4* evolutionary rate correlation (ERC) partners.
